# Pancreatic organogenesis mapped through space and time

**DOI:** 10.1038/s12276-024-01384-y

**Published:** 2025-01-08

**Authors:** Marissa A. Scavuzzo, Wojciech J. Szlachcic, Matthew C. Hill, Natalia M. Ziojla, Jessica Teaw, Jeffrey C. Carlson, Jonathan Tiessen, Jolanta Chmielowiec, James F. Martin, Malgorzata Borowiak

**Affiliations:** 1 Program in Developmental Biology, Baylor College of Medicine, Houston, TX USA; 2 Institute of Molecular Biology and Biotechnology, Adam Mickiewicz University, Poznan, Poland; 3 Center for Cell and Gene Therapy, Baylor College of Medicine, Texas Children’s Hospital, and Houston Methodist Hospital, Houston, TX USA; 4 Molecular and Cellular Biology Department, Baylor College of Medicine, Houston, TX USA; 5 Stem Cell and Regenerative Medicine Center, Baylor College of Medicine, Houston, TX USA; 6 Department of Molecular Physiology and Biophysics, Baylor College of Medicine, Houston, TX USA; 7 The Texas Heart Institute, Houston, TX USA; 8 Cardiovascular Research Institute, Baylor College of Medicine, Houston, TX USA; 9 McNair Medical Institute, Baylor College of Medicine, Houston, TX USA; 10 Present Address: Department of Genetics and Genome Sciences, Case Western Reserve University, Cleveland, OH USA

**Keywords:** Differentiation, Transgenic organisms

## Abstract

The spatial organization of cells within a tissue is dictated throughout dynamic developmental processes. We sought to understand whether cells geometrically coordinate with one another throughout development to achieve their organization. The pancreas is a complex cellular organ with a particular spatial organization. Signals from the mesenchyme, neurons, and endothelial cells instruct epithelial cell differentiation during pancreatic development. To understand the cellular diversity and spatial organization of the developing pancreatic niche, we mapped the spatial relationships between single cells over time. We found that four transcriptionally unique subtypes of mesenchyme in the developing pancreas spatially coordinate throughout development, with each subtype at fixed locations in space and time in relation to other cells, including beta cells, vasculature, and epithelial cells. Our work provides insight into the mechanisms of pancreatic development by showing that cells are organized in a space and time manner.

## Unique mesenchyme subtypes guide pancreas cell arrangement

This study explores how different types of cells, called mesenchyme, help form the pancreas during development. Researchers used various techniques to study mouse embryos and human fetal tissue. They identified several subtypes of mesenchyme in the developing pancreas and found that these subtypes are not randomly distributed; instead, they occupy specific locations. The study involved analyzing single-cell RNA sequencing data (a method to study gene expression in individual cells) and using advanced imaging techniques to map the positions of these cells. The researchers discovered that different mesenchyme subtypes have unique roles, such as supporting blood vessel formation or nerve development. These findings suggest that understanding mesenchyme organization could improve regenerative medicine approaches for diseases like diabetes. Future research may explore how these cells interact with other pancreatic components over time. This summary was initially drafted using artificial intelligence, then revised and fact-checked by the author.

## Introduction

Organ formation relies on the coordinated and dynamic interactions of a diverse repertoire of cell types to achieve precise cellular composition and architecture. As development must occur in a systematic manner, this implies that cells are geometrically synchronized with one another during organogenesis. The signaling and structural interactions between the developing pancreatic epithelium and the surrounding pancreatic niche are required for the expansion, reorganization, and differentiation of pancreatic cells. However, it is not known whether pancreatic niche cells randomly coalesce or if they follow a specific coordinate system in the developing pancreas.

The most abundant component of the niche is the mesenchyme. The contribution of the mesenchyme to proper pancreatic development has been recognized for decades^1^. However, how the mesenchyme regulates pancreatic differentiation is still under investigation. The early mesenchyme acts to prevent precocious differentiation of endocrine cells, keeping progenitors in a proliferative, multipotent state before initiation of branching^2,3^, with later mesenchyme exhibiting a positive effect on both exocrine and endocrine differentiation^4–8^. The mesenchyme in close proximity to epithelial cells promotes their differentiation into the exocrine lineage, whereas the mesenchyme at further distances fosters endocrine development^9,10^, suggesting that different subtypes of mesenchyme exist in the developing pancreas and that these different subtypes might be spatially organized.

In addition to mesenchymal cells, the niche also consists of endothelial cells and innervating neurons. Embryonic endothelial cells are required for endocrine differentiation and repression of epithelial proliferation and branching, whereas the adult vasculature forms close associations with endocrine cells for proper islet physiology through the exchange of nutrients and hormones^11–13^. Similarly, innervation of noradrenergic neurons is also necessary for islet physiology. Loss of this innervation in embryogenesis leads to developmental defects manifested by disrupted islet architecture^14,15^. Recent studies have shown that the pancreatic mesenchyme is molecularly heterogeneous^16,17^; however, whether different subtypes of mesenchyme are spatially arranged to differentially influence epithelial fate or niche organization is unclear.

As evidence continues to support the role of the pancreatic niche in modulating epithelial differentiation, we hypothesized that heterogeneous mesenchymal subtypes group in specific locations throughout pancreatic development. Through the application of a variety of techniques, we show that diverse mesenchyme subtypes migrate to and maintain precise coordinates in the developing pancreas.

## Materials and methods

### Animals

The animal studies were approved by the Baylor College of Medicine Institutional Animal Care and Use Committee and by the Local Ethical Committee for Animal Research, decision no. 46/02/2020 at Adam Mickiewicz University. All the mice were of mixed background and were housed at the Baylor College of Medicine Animal Facility. Nkx2-5-cre mice were originally generated by Dr. Richard P. Harvey^18^ and obtained from Dr. Daryl Armstrong Scott, Wt1-cre^ERt2^ mice were originally generated by Dr. William T. Pu^19^ and obtained from Dr. James F. Martin, Pdgfra-cre^ERT^ mice were originally generated by Dr. Dwight E. Bergles^20^ and obtained from The Jackson Laboratory (stock# 018280), Sun1-sfGFP was originally generated by Dr. Jeremy Nathans^21^ and obtained from Dr. James F. Martin, and ROSAmTmG was originally generated by Dr. Liqun Luo^22^ and obtained from The Jackson Laboratory (stock# 007676). The mice were housed at 22–24 °C with a 12 h light/12 h dark cycle with standard chow (Lab Diet Pico Lab 5V5R, 14.7% calories from fat, 63.3% calories from carbohydrates, 22.0% calories from protein) and water provided ad libitum. Genotyping was performed with the HotStart Mouse Genotyping Kit with its recommended PCR setup (KAPA Biosystems, USA). To genotype Nkx2-5-cre mice, three primers were used (F-GATTAGCTTAAGCGGAGCTGG, R1-GTTCTGGAACCAGATCTTGAC, and R2-GCCGCATAACCAGTGAAACAG), which yielded one band at 358 bp in wild-type mice, whereas heterozygous mice yielded two bands at 358 bp and 481 bp. To genotype the Wt1-cre^ERt2^ mice, four primers were used (Cre1-TGAAACAGGGGCAATGGTGCG, Cre2-CGGAATAGGTATGGGGGGCTCAG, Common1-GGCTTAAAGGCTAACCTGGTGTG, and Common2-GGAGCGGGAGAAATGGATATG), yielding one internal band at 374 bp and a Cre band at 200 bp. To genotype B6N.Cg-Tg(Pdgfra-cre/ERT)467Dbe/J mice, four primers were used (TransgeneF-TCAGCCTTAAGCTGGGACAT, CreR- ATGTTTAGCTGGCCCAAATG, Common1-CTAGGCCACAGAATTGAAAGACT, and Common2-CTAGGCCACAGAATTGAAAGATCT), yielding one internal positive control band at 324 bp and a transgene band at 492 bp. To genotype Sun1-GFP mice, three primers were used (S1-CATAGTCTAACTCGCGACACTG, S2-GCACTTGCTCTCCCAAAGTC, and S3-GTTATGTAACGCGGAACTCC), yielding one wild-type band at 557 bp or a mutant band at 300 bp. To genotype ROSA-mTmG mice, three primers were used (common F-CTCTGCTGCCTCCTGGCTTCT, R-CGAGGCGGATCACAAGCAATA, and CAG R-TCAATGGGCGGGGGTCGTT) with a 58 °C annealing temperature, yielding 320 bp wild-type and 250 bp EGFP-L10a bands.

To pulse-chase Wt1+ or Pdgfra+ cells, Wt1-cre^ERT2^:ROSAmTmG or Pdgfra-cre^ERT^:ROSAmTmG mice received a single intraperitoneal injection of tamoxifen (4-OHTm, Sigma-Aldrich, USA, #T5648, dissolved in corn oil) at 6 mg/40 g of body weight at embryonic day 10.5 (e10.5) or e12.5. The pancreas was analyzed 48–96 h later or in 8-month-old mice. For all the experiments, both male and female embryos were analyzed.

### 2D tissue collection, immunostaining, and confocal microscopy

The whole pancreas was fixed in 4% paraformaldehyde for 2‒16 h, washed with PBS, incubated in 30% sucrose overnight at 4 °C, and embedded in O.C.T. compound for sectioning and subsequent immunofluorescence staining. The nonspecific binding of the antibodies was blocked by a 30 min incubation with blocking solution (5% normal donkey serum in PBST) at RT. The primary antibodies were incubated in blocking solution for 16 h at 4 °C with shaking, and then the cells were washed three times with PBST for 10 min. The secondary antibodies were conjugated with the appropriate Alexa Fluor Dye (Jackson ImmunoResearch Europe, UK), diluted with blocking solution, incubated with the samples for 1 h at RT, and then washed three times with PBST. Nuclei were stained with DAPI (Invitrogen, USA). The slides were mounted in Fluoromount G (SouthernBiotech, USA), covered with coverslips, and sealed with nail polish. All primary antibodies and dilutions are listed in Supplementary Table 1. Imaging was performed on Zeiss 710 (Zeiss, Germany) and Nikon A1Rsi (Nikon, Japan) confocal microscopes.

### 3D tissue clearing, immunostaining, and light-sheet microscopy

The upper gastrointestinal region of embryonic mice was dissected from the stomach to the duodenum, including the spleen and pancreas, to ensure that the pancreatic tissue and surrounding mesenchyme were included. The tissues were then fixed overnight in 4% paraformaldehyde at 4 °C before being washed with PBS. To obtain optically clear tissue, the X-CLARITY tissue clearing system (Logos Biosystems, South Korea) was used without the initial incubation in hydrogel solution and proceeding directly to 3 h of incubation for hydrogel infusion and polymerization at 37 °C and −90 kPa. Next, the tissues were placed on a rocker for 2–3 min before being washed in 1x PBS and then placed in a mouse brain holder for immersion into the X-CLARITY apparatus. The tissues were placed in electrophoretic tissue clearing solution with a 1.2 A current at 37 °C with the solution flowing through the pump at 30 rpm for 2–3 h until the tissue became translucent. The tissues were then washed 5‒6 times in 1x PBS and incubated overnight while rocking in 1x PBS to remove residual SDS.

For antibody labeling, the tissues were incubated in 5% donkey serum in 1x PBST with primary antibodies at room temperature for 4 days (Supplementary Table 1). The tissues were washed for 2–3 h three times in 1x PBST before being incubated in secondary antibody solution in 5% donkey serum/1x PBST for 4 days at room temperature while shielded from light. After this incubation, the tissues were washed twice in 1x PBST for 2–3 h with rocking at room temperature before being stained with DAPI (Invitrogen). After two rinses in 1× PBS, the tissues were incubated in sRIMS media (70% sorbitol in 0.02 M phosphate buffer, pH 7.5) on a rocker at room temperature for 1 h before being embedded in 1% agarose in sRIMS in insulin syringes and imaged in sRIMS with the Lightsheet Z.1 (Zeiss) microscope at 5× with dual side fusion.

### Immunostaining and ethical approval of human developing pancreatic tissue

Human pancreas sections were processed at Baylor College of Medicine, Houston, TX (USA), with IRB-3097 approval granted to Malgorzata Borowiak. The donor identities were encrypted, and the data were analyzed anonymously. Human 10.6-, 13-, 16.3- and 20-week fetal pancreas samples were fixed in 4% paraformaldehyde/PBS for 4 h, washed with PBS, soaked in 30% sucrose, and embedded in TissueTek (Sakura Finetek, Netherlands). Sections (12 μm thick) were cut onto Superfrost Plus-coated glass slides and stored at −80 °C. Sections were stained by the same protocol used for frozen mouse sections.

### Spatial mapping of pancreatic cell types

Spatial mapping was carried out with Imaris software, with 3D images taken on a Zeiss Lightsheet Z.1. The segmentation of cells positively stained for markers was performed with Imaris with Spots (nuclear, Wt1) or Surfaces (cytoplasmic/membrane) functions. The Spots/Surfaces functions included thresholding for size and intensity (based on either absolute intensity or background subtraction, depending on the marker and image quality). Touching objects were segmented with the Region Growing function with a quality threshold. The positions of the cells were marked to measure their X, Y, and Z coordinates on a 3D grid and exported for each individual marker for further analysis with R. X, Y, and Z coordinates were then used to determine the Euclidean three-space distance through the equation (*d)* = $\sqrt{{\left({x}_{2}-{x}_{1}\right)}^{2}+{\left({y}_{2}-{y}_{1}\right)}^{2}+{\left({z}_{2}-{z}_{1}\right)}^{2}}$ with the rdist function in R. Euclidean three-space distance measurements were averaged and plotted or shown as density plots to visualize the relationships between cells as the number of cells spanning different distances between each other. Positions were also used for spatial mapping, with each X, Y, and Z coordinate used for orthogonal linear transformation to plot principal components, projecting the 3D relationships of cell populations in 2D space.

Alternatively, for complex structures formed by cells of the same type—i.e., Cdh1+ pancreatic epithelium, CD31+ endothelial network, and TH+ neural processes—for which segmentation to individual cells is computationally more challenging and might not be so precise, we used another method that was successful. A single surface was created instead of individual cell segmentation, which was used to compute a new channel (designated DT) with the Distance Transformation function in Imaris (Oxford Instruments, UK). The intensity values of the DT channel indicate the shortest distance to the computed surface; thus, for each identified cell of the other type (e.g., Wt1+), the distance to the surface (Cdh1 in this case) was equal to the DT channel intensity at the 3D position of the cell. The DT channel value for each cell was then plotted with R.

### Analysis of single-cell RNA sequencing data

Mesenchymal clusters were defined by Seurat^23^ analysis in R as described in Scavuzzo et al.^16^ (GEO GSM2689399 and GSM2689400 datasets for e14.5 and e16.5, respectively). Briefly, 15,228 single-cell transcriptomes were derived from 39 e14.5 pancreata from 3 litters, revealing 26 clusters from the top 22 principal components and 7 mesenchyme-associated clusters. Next, subclustering was performed with Seurat to subset 6,637 cells belonging to the Mes1, Mes2, Mes3, Mes4, MesO1, MesO2, and PaSC clusters, with the original identities stored as metadata. The cells were subclustered with batch correction by Harmony^24^, and then UMAP^25^ feature plots were created by Seurat. For pseudotemporal analysis, the normalized data from the indicated clusters calculated in Seurat were then passed directly into Monocle3^26^. The calculated pseudotime values were then inserted into Seurat object for visualization. Interactome analysis was performed with scRNA-seq data uploaded into the FANTOM5 database^27^ to determine the connectome from the ligand:receptor pairing and visualized with the *Connectome* R package^28^, filtering datasets for min.pct = 0.2 and max.p = 0.00001. The data are available as Dataset 1.

Human fetal pancreas PCW7-11 datasets (OMIX001616), created by Ma et al.^29^, were directly imported into Seurat, followed by subclustering of the mesenchyme and further analysis in Seurat and Monocle3. The human PCW12–20 fetal pancreas scRNA-Seq (GSE197064 dataset) and spatial transcriptomics (GSE197317 dataset) datasets created by Olaniru et al.^30^ were initially processed with 10x CellRanger and SpaceRanger tools, respectively, and further analyzed with Seurat. Stacked bar plots of population proportions were created with dittoSeq^31^. Spatial data were processed and then loaded per slice before using SCTransform and PCA to normalize the data. Slices from each timepoint were merged and corrected for batch effects with Harmony before calling clusters and projecting the UMAP of the data.

### Flow cytometry

The cell samples were pipetted through 70 μm cell strainers immediately before use to prevent clotting. For cell sorting, a BD FACSAria II instrument (BD Biosciences, USA), with 70-μm nozzle and 70-p.s.i. settings were used with FACSDIVA software (BD Biosciences), and the cells were sorted into a 1.5-ml Eppendorf tube containing sorting medium, consisting of DMEM/F12 supplemented with 20% FBS, penicillin–streptomycin and nonessential amino acids (all Thermo Fisher Scientific, USA). The sorted cells were then pelleted, the media was removed, and the cells were then suspended in 1 ml of TRIzol (Thermo Fisher Scientific) and immediately flash-frozen with liquid nitrogen.

### Total RNA extraction, library preparation, and sequencing

FAC-sorted cells were collected in TRIzol and snap-frozen. Total RNA was extracted from cells using the RNeasy Micro Kit (cat. no. 74004, Qiagen, Germany). RNA integrity (RIN ≥ 8.0) was confirmed with the High Sensitivity RNA Analysis Kit (DNF-472-0500, Agilent Tech. (formerly AATI), USA) on a 12-Capillary Fragment Analyzer. Illumina sequencing libraries with 8-bp single indices were constructed from 10 ng of total RNA with the Trio RNA-Seq System (0507-96, NuGEN, UK). The resulting libraries were validated with the Standard Sensitivity NGS Fragment Analysis Kit (DNF-473-0500, Agilent) on a 12-Capillary Fragment Analyzer. Equal concentrations (2 nM) of libraries were pooled and subjected to paired-end (2 × 75) sequencing of approximately 40 million reads per sample with the High Output v2 kit (FC-404-2002, Illumina, USA) on a NextSeq 500 (Illumina) following the manufacturer’s instructions.

### Ultra-low-input RNA-seq of the Nkx2-5+ mesenchyme subpopulation

FAC-sorted subpopulations were collected, and total RNA was extracted as outlined above. RNA integrity (RIN ≥ 8.0) was confirmed with the High Sensitivity RNA Analysis Kit (DNF-472-0500, Agilent) on a 12-Capillary Fragment Analyzer. cDNA synthesis was performed with the SMART-Seq Ultra Low Input RNA Kit for Sequencing (cat. no. 634889, Takara Bio, Japan) from approximately 250 pg total RNA. cDNA was validated with the High Sensitivity NGS Fragment Analysis Kit (DNF-474-0500, Agilent) on a 12-Capillary Fragment Analyzer. Quantification was determined with the Quant-iT dsDNA Assay Kit, high sensitivity (cat. no. Q33120, Thermo Fisher), and 100 pg of cDNA was tagmented and ligated with the Nextera XT DNA Library Kit (cat. no. FC-131-1024, Illumina) at ½ volumes to produce sequencing libraries. The resulting libraries were validated with the High Sensitivity NGS Fragment Analysis Kit on a 12-Capillary Fragment Analyzer and quantified with the Quant-iT dsDNA Assay Kit, high sensitivity. Equal concentrations (2 nM) of libraries were pooled and subjected to paired-end (2×75) sequencing of approximately 40 million reads per sample with the High Output v2 kit (FC-404-2002, Illumina) on a NextSeq500 (Illumina) following the manufacturer’s instructions.

### Statistical analysis

P values were calculated as indicated in the figure legends by two-sided Student’s *t* tests unless otherwise specified. The data are presented as the means ± SEMs, and the following symbols are used to represent p values: *p < 0.05, **p < 0.01, ***p < 0.005, and ****p < 0.001. N represents the number of independent experiments.

## Results

### Diverse subtypes of the pancreatic mesenchyme exist in the murine embryonic pancreas

**Fig. 1:**
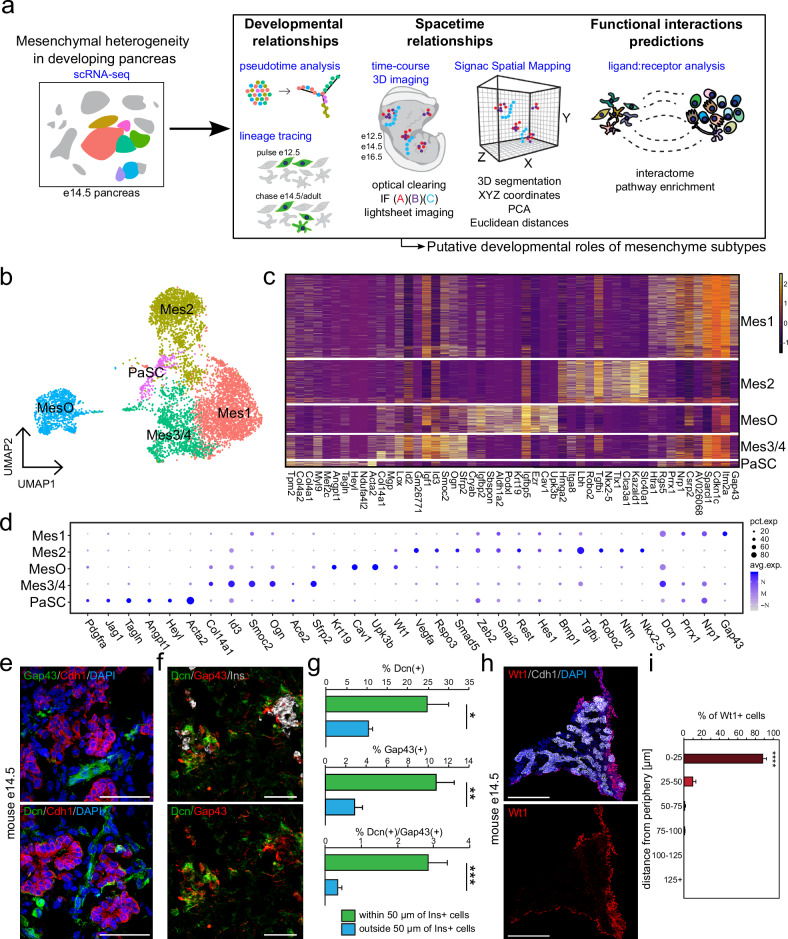
Mesenchymal subtypes in the developing pancreas exhibit distinct spatial distributions. **a** Schematic illustration of the experimental pipeline. We have subclustered mesenchymal cells from our previously published single-cell RNA sequencing^19^ dataset to further characterize specific mesenchymal subtypes by bioinformatic analyses and experimental validation, i.e., lineage tracing and 3D spatial mapping. **b** UMAP feature plot representation of 6,637 single mesenchymal cell transcriptomes grouped by graph-based clustering. **c** Heatmap of the top 10 variably expressed genes for each subcluster (x-axis) in single mesenchymal cells (y-axis). The expression ranges from low (dark purple) to high (yellow). **d** Dot plot showing select mesenchymal subtype marker genes, with denoted prevalence (i.e., % of cells within a cluster that expresses a marker; pct. exp., dot size) and average expression levels (avg. exp., dot color) for each subtype. **e** Immunostaining of the mouse e14.5 pancreas showing the developing pancreatic epithelium (Cdh1, red) and the expression of the mesenchyme subtype markers Gap43 (Mes1) and Dcn (Mes1, Mes3/4, and MesO) (both green). Nuclei are labeled with DAPI (blue). Scale bar = 50 μm. **f** Immunostaining of the mouse e14.5 pancreas showing developing pancreatic beta cells (Ins, white) and mesenchymal cells marked by Gap43 (red) and Dcn (green) in proximity. Nuclei are labeled with DAPI (blue). Scale bar = 50 μm. **g** Frequency distribution plots showing the percentages of Gap43+ (Mes1), Dcn+ (Mes1, Mes3/4, MesO) and double-positive Gap43+Dcn+ (Mes1) cells at specific binned proximities to Ins+ endocrine cells, corresponding to (**f**). *N* = 4 mice (e14.5), with 3–8 images quantified per animal. The error bars represent the SEM. *p < 0.05, **p < 0.01, ***p < 0.005. h. Immunostaining of the mouse e14.5 pancreas showing the epithelium (Cdh1+, white) and mesothelium (Wt1+ MesO, red). Nuclei are labeled with DAPI (in blue). Scale bars = 200 μm. **i** Frequency distribution plots showing the frequency of Wt1+ cells within a specific 25 μm binned distance to the e14.5 pancreas periphery, corresponding to (**h**). *N* = 3 mice. ****p < 0.001.

The pancreatic mesenchyme supports the development of epithelial cells during embryogenesis and the in vitro expansion of human stem cell-derived endoderm and pancreatic endocrine progenitors^5,32,33^. Our previous work and that of others identified the presence of multiple transcriptionally distinct mesenchyme subtypes during pancreatic development^16^ (Fig. 1a). From these single-cell RNA-sequencing analyses, we identified four mesenchyme subtypes (Mes1–4), two mesothelial subtypes (MesO1/2), and a cluster of pancreatic stellate cells (PaSCs). These clusters, containing 6,637 single-cell transcriptomes, were computationally selected for subclustering, after which redundant clusters were collapsed, leaving five transcriptionally distinct mesenchymal clusters: Mes1, Mes2, Mes3/4, mesothelial cells (MesO), and pancreatic stellate cells (PaSCs) (Fig. 1b). The most abundant mesenchyme subtype, Mes1, specifically expressed the neuron growth-associated gene *Gap43*, along with the angiogenesis- and migration-related genes *Nrp1* and *Dcn* (Fig. 1c, d**)**. In addition, Mes1 expressed *Prrx1*, a transcription factor that has been shown to regulate the vascularization of the lung^34,35^. Mes2, the second most abundant mesenchyme subtype, highly expressed a cohort of angiogenesis-related genes, including *Robo2, Vegfa, Vegfc, Rspo3, Nkx2-5, Ntm*, and *Tgfbi*, many of which function through regulation of the Wnt signaling or TGFb signaling pathways^36–40^ (Fig. 1c, d). MesO cells transcriptionally resembled the epicardium, with the enrichment of *Upk3b, Wt1, Cav1, Ezr, Krt19*, and *Aldh1a2*^41^. Finally, Mes3/4 and PaSCs were composed of the rarest subtypes of mesenchymal cells and expressed *Srfp2*, *Ace2*, and *Acta2*, respectively (Fig. 1c, d).

### Mesenchymal subtypes form neighborhoods with distinct spatial distributions

To further characterize the diversity of the e14.5 pancreatic mesenchyme, we confirmed the specificity of different mesenchyme subtype markers by first computationally quantifying their expression in each subtype (Supplementary Fig. 1a) before using flow cytometry to validate the abundance of each marker and determine the ability to detect each with an antibody (Supplementary Fig. 1b). From this analysis, we validated that the percentage of cells expressing Ace2, Ntm, and Nkx2-5 was almost equal (within 1.5%) by flow cytometry and single-cell RNA sequencing. We confirmed that the subtypes coexpressed the mesenchymal marker vimentin (Vim) but not the epithelial marker E-cadherin (Cdh1; Supplementary Fig. 1c–f).

Importantly, through immunofluorescence staining, we observed that the mesenchyme subtypes were not randomly arranged throughout the developing pancreas. For example, we found the Mes1 subtype, which highly expressed *Dcn* and *Gap43*, next to the pancreatic epithelium (marked by Cdh1, Fig. 1e). Furthermore, we found Gap43+/Dcn+ Mes1 cells spatially positioned near Ins+ cell clusters, with Gap43+, Dcn+, or double-positive cells rarely found more than 50 µm from Ins+ cells (Fig. 1f, g). This subtype of mesenchyme, Mes1, was enriched for genes associated with cell migration and epithelial morphogenesis, which are features of endocrine cells as they migrate and cluster into islets (Supplementary Fig. 2a). Other mesenchymal subtypes were enriched for genes associated with other processes (Supplementary Fig. 2b–e), including mesenchyme development and epithelial differentiation in MesO and enrichment for terms related to vascular development, including blood vessel morphogenesis, angiogenesis, vasculature development, and blood vessel remodeling in Mes2. Finally, PaSCs were enriched for expected functions, including response to virus and wounding as well as cell division^42^ (Supplementary Fig. 2e).

The transcription factor Wt1 is a marker of the peripheral pancreatic mesenchyme^17,43^, which we found to be expressed in the MesO mesothelial cluster and to a much lower extent in Mes2. Analysis of the Wt1+ MesO subtype through immunostaining revealed a peripheral localization in the developing mouse pancreas (Fig. 1h). We computationally segmented the pancreatic sections into 25-micron bins to assess the distribution of Wt1+ MesO cells in the pancreas. We found that a significant majority of these cells were within 25 microns of the periphery (Fig. 1i), suggesting that this subtype contributes to the mesothelium. Together, the GO and immunofluorescence analyses suggest the spatial and functional diversity of different mesenchyme subtypes.

### The peripheral mesothelium gives rise to other mesenchymal subtypes

**Fig. 2:**
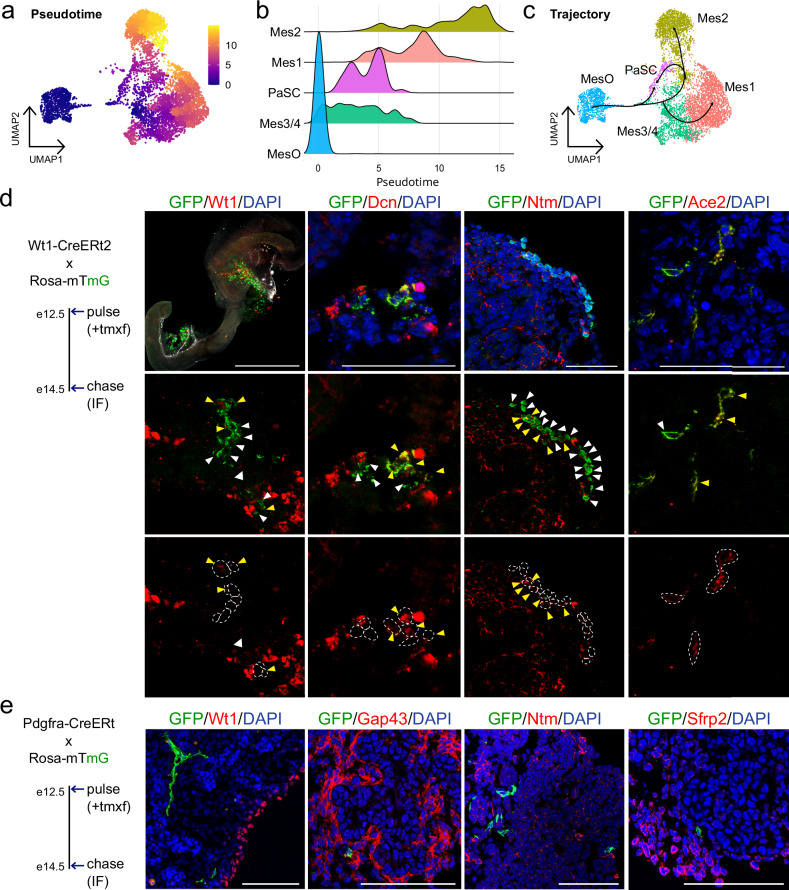
The mesothelium gives rise to other pancreatic mesenchyme cells during embryogenesis. **a** UMAP plot showing the pseudotime values of single e14.5 mesenchymal cells calculated by Monocle3. **b** Density plots showing the distribution of mesenchymal cells over pseudotime. **c** UMAP plot showing the inferred trajectories based on Monocle3 predictions. **d** *Left:* Schematic for lineage tracing of Wt1-Cre^ERt2^ mice crossed with Rosa-mTmG. Mice were pulsed with tamoxifen (tmxf) to label Wt1+ cells and their progeny with GFP at e12.5, and tissue was collected at e14.5 for immunofluorescence (IF) analysis. *Right:* Immunostaining showing GFP+ cells (e12.5 Wt1+ progeny, green) overlapping in expression with Wt1+ (MesO), Dcn+ (Mes1, Mes3/4, MesO), Ntm+ (Mes2), and Ace2+ (Mes4) cells in the red channel. Yellow arrows show overlapping GFP+ and marker-positive cells, white arrows show GFP+ cells alone, and circles in the bottom panel mark GFP+ cells. DAPI (blue) was used to stain the nuclei. Scale bars = 50 μm. **e** *Left:* Schematic for lineage tracing of Pdgfra-Cre^ERt^ mice crossed with Rosa-mTmG, analyzed as in B. *Right:* Immunostaining showing GFP+ cells (e12.5 Pdgfra+ progeny, green) that lacked coexpression with Wt1+ (MesO), Gap43+ (Mes1), Ntm+ (Mes2), and Sfrp2+ (Mes3/4) cells (red). DAPI (blue) was used to stain the nuclei. Scale bars = 50 μm. See also Supplementary Fig. 3b.

In early pancreatic development, FGF signaling is essential for the proliferation and expansion of the primordial pancreas^44^. The MesO subtype, enriched for signals associated with mesenchyme development (Supplementary Fig. 2d), had highly enriched expression of FGF ligands (Supplementary Fig. 2f). In particular, *Fgf9* was significantly enriched; loss of FGF9 leads to reduced development of the pancreatic mesenchyme^45^. Together, these findings led us to hypothesize that MesO gives rise to other mesenchymal cell types. To understand the transcriptional relationships among mesenchymal cells, we performed trajectory analysis with Monocle 3. This analysis positioned MesO cells as the earliest population along the pseudotime trajectory, followed by Mes3/4 cells and PaSCs. In contrast, Mes1 and Mes2 were inferred to occupy later positions in the trajectory (Fig. 2a, b). Trajectory inference suggested that Mes3/4 serves as a branching point for either Mes1 or Mes2 branches, whereas PaSCs could potentially arise from both Mes3/4 and Mes2 (Fig. 2c).

To determine whether these transcriptional relationships predict computationally represented developmental lineages, we crossed Wt1-cre^ERt2^ mice^19^ with Rosa-mTmG mice^22^ to mark all Wt1 progeny with GFP upon administration of tamoxifen. As *Wt1* is expressed in MesO (Fig. 1c, d), after Cre recombination, GFP marks MesO cells as well as their progeny. After the mice were pulsed with tamoxifen at e12.5, we collected tissue 48 h later at e14.5 to analyze their lineage (Fig. 2d). We found GFP+ cells derived from Wt1+ that no longer expressed Wt1 but developed into Wt1-/Dcn1+, Wt1-/Ntm+, or Wt1-/Ace2+ cells. This finding indicates that Wt1+ cells can give rise to different mesenchyme subtypes belonging to both developmental branches, as predicted by pseudotime. The Monocle3 trajectory inference analysis suggested that PaSCs populated intermediate pseudotime values, indicating that they occupy side branches diverging from either Mes3/4 or Mes2 trajectories. Therefore, we next checked whether PaSCs were limited in their potential or if they had the capacity to give rise to different mesenchyme subtypes. To do this, we crossed a separate mouse line, Pdgfra-cre^ERt2 20^, with Rosa-mTmG to mark PaSCs and their progeny with GFP upon tamoxifen administration and used the same experimental timeline as for Wt1-cre^ERt2^ mice (Fig. 2e). We collected pancreata from e12.5-pulsed mice in adulthood and found that the GFP+ progeny of e12.5 PaSCs did not express Pdgfrb (pericyte marker), but some GFP+ cells coexpressed Acta2 (vascular smooth muscle cell marker) and Gfap (adult PaSC marker) (Supplementary Fig. 3a). These findings suggest that e12.5 PaSCs do not contribute significantly to pericytes present in adult mice but that some vascular smooth muscle cells and adult PaSCs are progeny of e12.5 PaSCs. At e14.5, we observed overlap of GFP with some Ace2+ and Dcn+ cells (Supplementary Fig. 3b), which are markers that are expressed by some PaSCs and mesenchyme subtypes. Importantly, we found no overlap of GFP with staining for Wt1, Gap43, Ntm, or Sfrp2 (Fig. 2d). This finding suggests that Wt1+ MesO lines the periphery of the pancreas and that Wt1+ cells, but not Gfap+ cells, can develop into other mesenchymal subtypes.

To determine whether the mesenchyme populations at the terminal branches of pseudotime remain static or continue to change over time, we computationally selected cells from branch end2 (Mes1 and Mes3/4) representing e14.5 before merging them with e16.5 Mes1 and Mes3/4 cells from single-cell RNA sequencing^19^. Unsupervised clustering revealed that mesenchyme subtypes grouped separately from each age group, suggesting that cells from each age point were transcriptionally unique (Supplementary Fig. 3c). To investigate branch end1 (Mes2), which consisted of fewer cells, we used Nkx2-5 reporter mice (Nkx2-5-cre;Sun1-eGFP)^18^ at e14.5 and e16.5 to isolate Nkx2-5+ cells before using Smart-seq2 for high-depth sequencing and observed widespread transcriptional changes over these two timepoints (Supplementary Fig. 3d–f), suggesting that Mes2 changes over time. Next, we immunostained tissue throughout the secondary transition of pancreatic development when massive spatial reorganization is occurring for the markers of the mesenchyme subtypes we identified (Supplementary Fig. 3g). We found that these markers were expressed at each timepoint and appeared to be spatially arranged relative to other cell types. This finding indicates that the transcriptional changes we observed over time may reflect changes in mesenchyme function over time as they mature rather than an instability of subtype identities. Thus, we hypothesized that mesenchymal cells follow a geometric coordinate system throughout development to spatially arrange themselves in specific relationships with other pancreatic cell types.

### Space-time analysis of pancreatic niche cells

The relationship between the peripheral mesothelium and other cell types prompted us to ask whether mesenchyme subtypes may occupy precise locations with respect to one another. To assess the 3D spatial organization of mesenchyme subtypes in three dimensions (3D) over time, we dissected and optically cleared the mouse gastrointestinal (GI) region at three timepoints (e12.5, e14.5, and e16.5) spanning the secondary transition of pancreatic development when robust architectural changes occur. In our preparations for spatial mapping, we included the stomach, intestine, spleen, and pancreas to prevent loss of any of the surrounding connective tissues while keeping the adjacent tissues intact as landmarks (Supplementary Fig. 4a, b). Optically cleared tissues were then immunostained with specific cluster markers before 3D imaging with light-sheet microscopy to assess the localization of mesenchyme subtypes within the developing pancreas.

Our single-cell RNA-sequencing analysis revealed the coexpression of *Gap43, Dcn*, and *Epha4* transcripts throughout the Mes1 mesenchyme subtype (Supplementary Fig. 4c). Concurrent with the single-cell RNA sequencing results, we detected the coexpression of Gap43 and Epha4 in the e14.5 pancreas (Supplementary Fig. 4d). We observed that Epha4 formed a web-like network around the pancreas (Supplementary Fig. 4e) and that the structure and patterning of Dcn were close to the embryonic vasculature (Supplementary Fig. 4f).

### Spatial mapping of the mesothelium over time in relation to epithelial and beta cells

We hypothesized that mesenchyme subtypes could be spatially arranged in the developing pancreas. Therefore, we set out to define the neighborhood dynamics of mesenchyme subtypes in relation to other cell types, including nerves, blood vessels, beta cells, and the pancreatic epithelium.

**Fig. 3:**
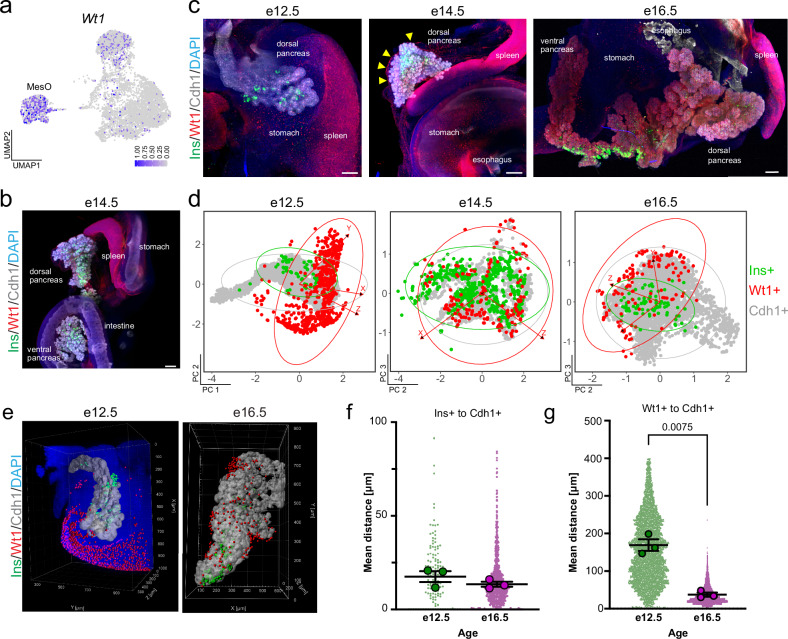
Space–time mapping of the pancreatic MesO. **a** Single-cell RNA-sequencing UMAP feature plot showing the expression of *Wt1*, with dark blue indicating high expression and gray indicating no expression. The MesO subtype is denoted. **b** Representative maximum-intensity projection light-sheet microscopy image of intact, optically cleared e14.5 pancreas and surrounding organs immunostained for the Wt1+ MesO marker (red), beta cells (Ins+, green), and epithelium (Cdh1+, gray). Nuclei are labeled with DAPI (blue). Scale bars = 200 μm. See also Supplementary Fig. 5b. **c** Representative maximum-intensity projection light-sheet microscopy images of intact, optically cleared e12.5, e14.5 and e16.5 mouse pancreata stained as in (**b**). In the e14.5 image, arrowheads point to the pancreatic MesO layer. Nuclei are labeled with DAPI (blue). Scale bars = 100 μm (e12.5), 200 μm (e14.5 and e16.5). See also Supplementary Fig. 5c. **d** Principal component analysis from X, Y, and Z position coordinates derived from an overlaid 3D grid. Each dot represents an individual cell, with similarly localized cells in closer proximity, whereas those at further distances are more separate within the plotted dimensions (PCs). Ellipses indicate regions including 95% of the cells within a cell type population. **e** 3D rendering of MesO (red), beta cells (green) and epithelium (white) in the whole region of the e12.5 and the middle region of e16.5 mouse pancreata immunostained for Wt1, Ins and Cdh1, respectively, captured by light-sheet microscopy. In e12.5, the DAPI staining signal (blue) is shown to visualize the nuclei. **f**, **g** Plots of Euclidean three-space distances between Ins+ or Wt1+ cells and the Cdh1+ epithelium in e12.5 and e16.5 mouse pancreata. Large dots represent the mean shortest distance between individual cells per embryo, whereas small dots represent the shortest distance measurements for individual cells. The bars indicate the means and SEMs of biological replicates. *P* values were determined by Brown-Forsythe and Welch one-way ANOVA tests with Dunnett’s T3 multiple comparisons tests and are shown for statistically significant differences. *N* = 3 embryos and for individual cells: (*Ins+ to Cdh1*+) *N* = 128 for e12.5 and 865 for e16.5, and (*Wt1+ to Cdh1*+) *N* = 6,100 for e12.5 and 4,606 for e16.5.

The Wt1+ peripheral pancreatic mesothelium provided signals that instructed the proper migration of endocrine progenitors into the surrounding milieu^10^ (Fig. 3a). After 3D imaging, we confirmed that Wt1+ cells line the periphery of the pancreas, intestine, and stomach and observed a strong signal throughout the spleen **(**Fig. 3b and Supplementary Fig. 5b and Movie 1). Throughout the second transition of pancreatic development, we assessed the space–time relationship of MesO cells to epithelial and beta cells (Fig. 3c, Supplementary Fig. 5a–d). This finding revealed the proximity of beta cells (Ins) to the epithelium (Cdh1), whereas Wt1+ MesO resided apart throughout all three timepoints (Fig. 3c, Supplementary Fig. 5a–d).

To visualize 3D spatial relationships, we utilized spatial distance mapping. First, we generated 3D renderings of light-sheet images. Next, we measured the 3D spatial coordinates of each cell and transformed them into 2D space with principal component analysis (PCA). This resulting 2D representations, termed spatial distance mapping (Fig. 3d), effectively cluster neighboring cells closer together while placing cells that are more distant further apart. Through this approach, we confirmed that the majority of Wt1+ cells encircle epithelial and beta cells from e12.5 to e16.5 (Fig. 3d). We then used 3D renderings of cells in the pancreas during the secondary transition (e12.5 to e16.5; Fig. 3e) for quantitative space–time analyses. We found that Ins+ beta cells retained a fairly similar distance to Cdh1+ epithelium over time (Fig. 3f). When we examined the space–time relationships between Wt1+ cells and Cdh1+ cells, we found that MesO was significantly closer to the epithelium at e16.5 than at e12.5 (Fig. 3g). This concurs with previous findings that there is a decrease in the volume of the pancreatic mesenchyme as the tissue develops and matures during embryogenesis, bringing the outer lining closer to the epithelium^32,46^. Beta cells delaminate from the epithelium and cluster together into endocrine islets throughout the secondary transition^47^; this means that Ins+ cells can be expected to be found near other Ins+ cells. Next, we mapped the 95% confidence intervals of spatial relationships between cell types at different timepoints, and for the Wt1-Cdh1 and Wt1-Ins pairs, we found no significant differences compared with the control Ins-Ins relationship (Supplementary Fig. 5e). This finding suggests that these cellular relationships, or the distances between these cells, are consistent between biological replicates and that cells can be expected to be found at specific locations throughout development. Overall, space–time mapping confirmed the tissue-wide spatial separation of Wt1+ MesO from the epithelium and endocrine cells, contrasting with the close association of beta-cells and their proximity to the parental epithelium.

### Mesenchymal space–time relationship with pancreas innervation

Space–time mapping analysis of Wt1+ peripheral mesothelium confirmed the robustness of methods for detecting spatial relationships and their dynamic changes over time. We next sought to determine the cellular relationships of other mesenchyme subtypes and niche cells. The Mes3/4 mesenchyme subtype was predicted to regulate neural innervation, with processes such as axon extension and guidance, neuron projection, and GDNF receptor signaling enriched by gene ontology (Supplementary Fig. 2c). In the adult pancreas, neurons are concentrated around islets^48^.

**Fig. 4:**
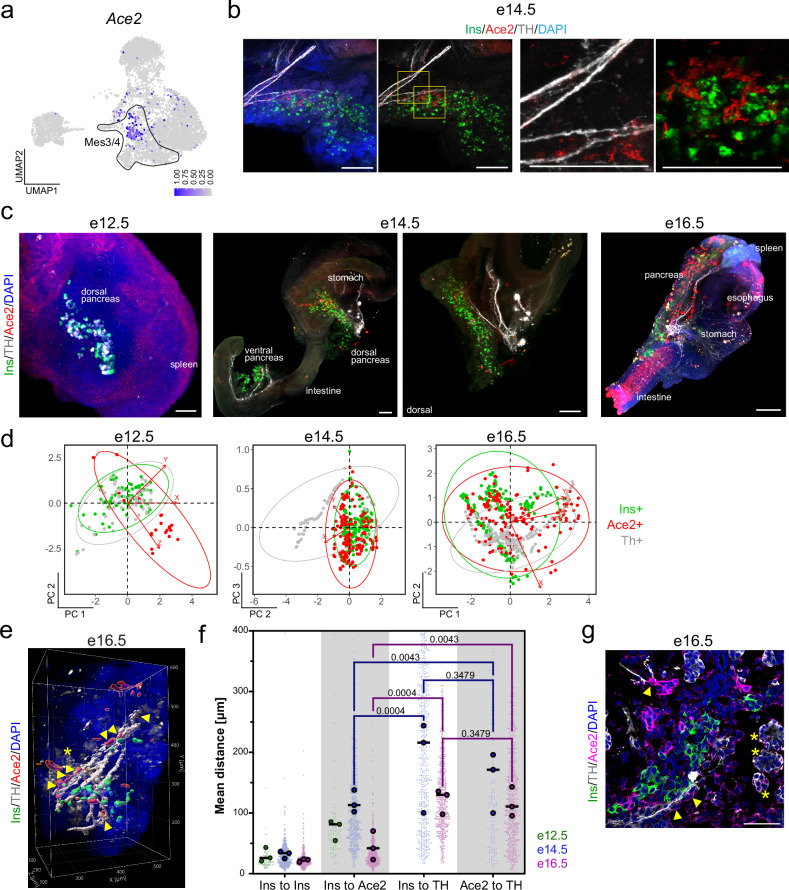
Space–time mapping of the Ace2+ mesenchyme relative to innervating neurons and beta cells. **a** Single-cell RNA sequencing UMAP feature plot showing the mesenchymal expression of *Ace2*, with dark blue indicating high expression and gray indicating no expression. The circle outlines Mes3/4. **b** Representative immunostaining images of the optically cleared 3D mouse e14.5 pancreas for beta cells (Ins, green), Ace2+ mesenchyme (red), neurons (TH, gray), and nuclei (DAPI, blue). Scale bars = 200 μm. **c** Representative maximum-intensity projection light-sheet microscopy images of intact, optically cleared e12.5, e14.5, and e16.5 mouse pancreata immunostained for Ace2+ mesenchyme (red), beta cells (Ins+, green), and sympathetic neurons (TH+, not present at e12.5). Nuclei are labeled with DAPI (blue). Scale bars = 100 μm (e12.5), 200 μm (e14.5), and 500 μm (e16.5). See also Supplementary Fig. 6b. **d** Principal component analysis from X, Y, and Z position coordinates derived from an overlaid 3D grid. Each dot represents an individual cell, with similarly localized cells in closer proximity, whereas those at further distances are more separate within the plotted dimensions (PCs). Ellipses indicate regions including 95% of the cells within a cell type population. **e** Example 3D rendering of the Ace2+ mesenchyme (red), Ins+ beta cells (green) and TH+ sympathetic neurons (white) in the middle region of the e16.5 mouse pancreas, as captured by light-sheet microscopy. TH+ staining indicates sympathetic neurons and alpha cells (asterisk-marked cells at e16.5). Arrowheads indicate Ace2+ cells close to neuronal processes. An asterisk indicates TH-expressing alpha cells. The DAPI staining signal (blue) is shown to visualize the nuclei. **f** Plots of Euclidean three-space distances between Ins+, Ace2+ cells, and TH+ neurons in e14.5 (left) and e16.5 (right) mouse pancreata. At e12.5, there are no TH+ neural processes. Large dots represent the mean shortest distance between individual cells per embryo, whereas small dots represent the shortest distance measurements for individual cells. The bars indicate the means and SEMs of biological replicates. P values were determined by Brown-Forsythe and Welch one-way ANOVA tests with Dunnett’s T3 multiple comparisons tests. *N* = 3 embryos and for individual cells: (*Ins+ to Ins*+*, Ins+ or TH*+) *N* = 675 for e14.5 and 600 for e16.5, and (*Ace2+ to TH*+) *N* = 170 for e14.5 and 692 for e16.5. **g** Representative confocal images of the mouse e16.5 pancreas immunostained for Ace2 (magenta), beta cells (Ins+, green), and TH+ sympathetic neurons (gray). Arrowheads indicate Ace2+ mesenchymal cells close to neuronal processes, and asterisks indicate TH+ alpha cells. Nuclei are labeled with DAPI (blue). Scale bar = 50 μm.

We used *Ace2*, which had an enriched RNA expression in a subpopulation of Mes3/4 cells (Fig. 4a), to map the location of Ace2+ mesenchymal cells in relation to neurons and beta cells. Using 3D imaging, we observed that Ace2+ cells localized near Ins+ beta cells in both the dorsal and ventral pancreas at e14.5 (Fig. 4b). We then performed whole-mount staining and light-sheet imaging of the e12.5-e16.5 pancreas (Fig. 4b, c, Supplementary Fig. 6a–d and Movie 2) and observed that the density of Ace2+ cells near beta cells increased with age. At e12.5, we did not detect neural processes stained by TH, and the cells staining positive with the TH antibody were in close relation to Ins+ cells (Fig. 4c, Supplementary Fig. 6a), which corroborates TH expression in pancreatic alpha cells^49^. At e14.5 and e16.5, we observed TH+ neural projections in the pancreas (Fig. 4c, Supplementary Fig. 6b–d). Using spatial mapping, we found that Ace2+ cells overlapped with Ins+ beta cells at e14.5–e16.5, but only a fraction of both cell types was in proximity to neuronal processes (Fig. 4d). We next measured the distances between every positively stained cell for Ins, Ace2 and TH in the pancreas, which revealed that beta cells and most Ace2+ mesenchyme cells were closer to each other than to nerves, mirroring the results shown through spatial mapping (Fig. 4e, f). Interestingly, at e16.5, we observed a small fraction (~7.5%) of Ace2+ cells adjacent to TH+ neurons (Fig. 4e–g). The 95% confidence intervals of the mapped distances again revealed that the cells appeared to be at fixed locations, with most notably, Ins+ cells mapped to the same location relative to the Ace2+ mesenchyme (Supplementary Fig. 6e). Together, these results reveal a close association between Ace2+ mesenchymal cells and beta cells during a stage in which nerves innervate the pancreas and islets cluster together.

### Mesenchymal space–time relationship with the pancreatic vasculature

**Fig. 5:**
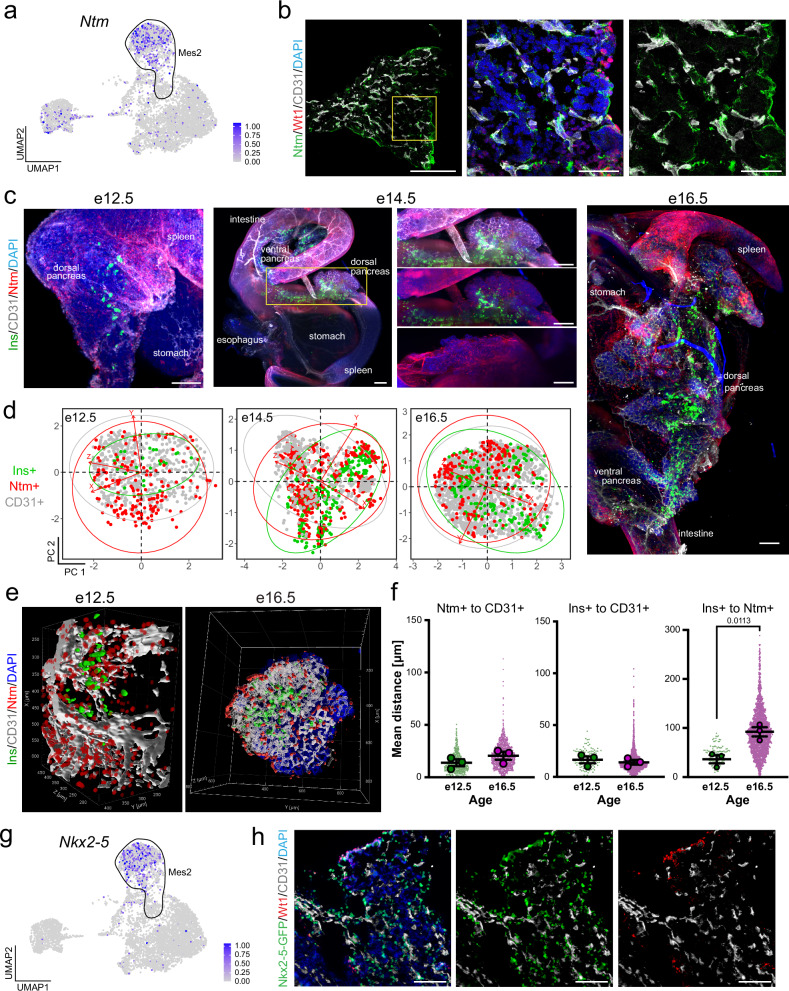
Spatial mapping of Mes2 relative to endothelial cells and beta cells. **a** Single-cell RNA-sequencing UMAP feature plot showing the expression of *Ntm*, with dark blue indicating high expression and gray indicating no expression. The Mes2 subtype is denoted. **b** Immunostaining of the mouse e14.5 pancreas showing the Mes2 population (Ntm+, green), MesO (Wt1+, red), and endothelium (CD31+, gray), with nuclei labeled with DAPI (blue). Scale bars = 200 μm (left-most image) and 50 μm (two right images). **c** Representative maximum-intensity projection light-sheet microscopy images of intact, optically cleared e12.5, e14.5, and e14.5 mouse pancreata immunostained for Mes2 (Ntm+, red), beta cells (Ins+, green), and endothelium (CD31+, gray). Nuclei are marked by DAPI in blue. Scale bars = 200 μm. **d** Principal component analysis from X, Y, and Z position coordinates derived from an overlaid 3D grid. Each dot represents an individual cell, with similarly localized cells in closer proximity, whereas those at further distances are more separate within the plotted dimensions (PCs). Ellipses indicate regions including 95% of the cells within a cell type population. **e** 3D rendering of Mes2 (Ntm, red), beta cells (Ins, green) and blood vessels (CD31, white) in the whole e12.5 and the middle region of e16.5 mouse pancreata immunostained for Ntm, Ins, and CD31, respectively, captured by light-sheet microscopy. In e16.5, the DAPI staining signal (blue) is shown to visualize the nuclei. **f** Plots of Euclidean three-space distances between Ntm+ or Ins+ cells and CD31+ endothelial cells or between Ins+ and Ntm+ cells in e12.5 and e16.5 mouse pancreata. Large dots represent the mean shortest distance between individual cells per embryo, whereas small dots represent the shortest distance measurements for individual cells. The bars indicate the means and SEMs of biological replicates. *P* values were determined by Brown-Forsythe and Welch one-way ANOVA tests with Dunnett’s T3 multiple comparisons tests and are shown for statistically significant differences. *N* = 3 embryos and for individual cells: (*Ntm+ to CD31*+) *N* = 857 for e12.5 and 1397 for e16.5, (*Ins+ to CD31+ and Ins+ to Ntm*+) *N* = 124 for e12.5 and 2207 for e16.5. **g** Single-cell RNA-sequencing UMAP feature plot showing the expression of *Nkx2-5*, with dark blue indicating high expression and gray indicating no expression. The Mes2 subtype is denoted. **h** Immunostaining of the Nkx2-5-GFP e14.5 mouse pancreas. Nkx2-5-GFP marks Mes2 (green), MesO marked by Wt1 (red), endothelial cells marked by CD31 (white), and nuclei marked by DAPI (blue). Scale bars = 100 μm.

We next applied space–time mapping to evaluate the relationships of Mes2 with beta cells and endothelial cells, as we found that Mes2 was enriched for endocrine cell development and that cell adhesion and beta cells were located proximal to the vasculature (Supplementary Fig. 2b). We observed the localization of beta cells (Ins+) and the vasculature (CD31+), finding, as expected, that beta cells nestled near endothelial cells^11–13^ (Supplementary Fig. 7a, b). Using *Ntm* as a marker of Mes2 (Fig. 5a), we found by high-resolution confocal microscopy the close association of Ntm+ Mes2 mesenchyme with CD31+ endothelial cells (Fig. 5b). We then conducted 3D analysis. Light-sheet imaging of e12.5–e16.5 pancreata (Fig. 5c, Supplementary Fig. 7c, d) revealed that Mes2 webbed throughout the developing e14.5–e16.5 pancreatic epithelium in a mesh-like network similar to that of the CD31+ vasculature (Fig. 5c, see the e14.5 close-up). We next determined the spatial arrangement of Mes2 cells in relation to endothelial cells and Ins+ cells throughout development. Spatial distance mapping suggested that some Ntm+ cells were closely juxtaposed with Ins+ beta cells and that both cell types were enveloped within a mesh of endothelial cells (Fig. 5d). However, these cell types also formed spatially separated clusters, which became more prevalent over time (Fig. 5d). We further confirmed through spatial distance measurements that Ntm+ and Ins+ cells remained closer to CD31+ vessels than to each other and that the distance between them increased from e12.5 to e16.5 (Fig. 5e–f). Confidence mapping of distance values revealed that the vasculature, marked by CD31, maintained fixed positions relative to Ins+ beta cells and the Ntm+ mesenchyme. However, there was no apparent spatial coordination between Ins+ beta cells and the Ntm+ mesenchyme (Supplementary Fig. 7e).

The *Nkx2-5* gene was also highly enriched and specific to Mes2 (Fig. 5g); thus, we used Nkx2-5 reporter mice to confirm the localization of Mes2 in relation to endothelial cells. We again observed a close association of Nkx2-5+ Mes2 with CD31+ endothelial cells (Fig. 5h). Pericytes are perivascular mesenchymal derivatives^50^, and through immunofluorescence staining, we investigated whether e14.5 Ntm+ and Nkx2-5+ cells colocalize with Pdgfrb, a marker of pericytes in the developing pancreas^50^. We found that some, but not all, Mes2 cells colocalized with Pdgfrb + , whereas Sfrp2+ Mes3/4 cells were more distant from Pdgfrb+ and CD31+ cells (Supplementary Fig. 7f). Interestingly, not all Pdgfrb+ cells colocalized with CD31+ vessels. Together, these results reveal that the Mes2 mesenchyme is a nonpericytic population that maintains spatial proximity to the forming vasculature while remaining distant from endocrine cell clusters.

### Mesenchyme subtypes are predicted to interact with different pancreatic cell types

The pancreatic niche influences the development of the pancreatic epithelium through a combination of paracrine cues and structural support^5^. However, whether subtypes of the mesenchyme specifically express these cues is not yet known. Gene ontology analysis revealed the enrichment of gene sets involved in specific functional processes, suggesting that mesenchyme subtypes are functionally specialized (Supplementary Fig. 2a–e).

**Fig. 6:**
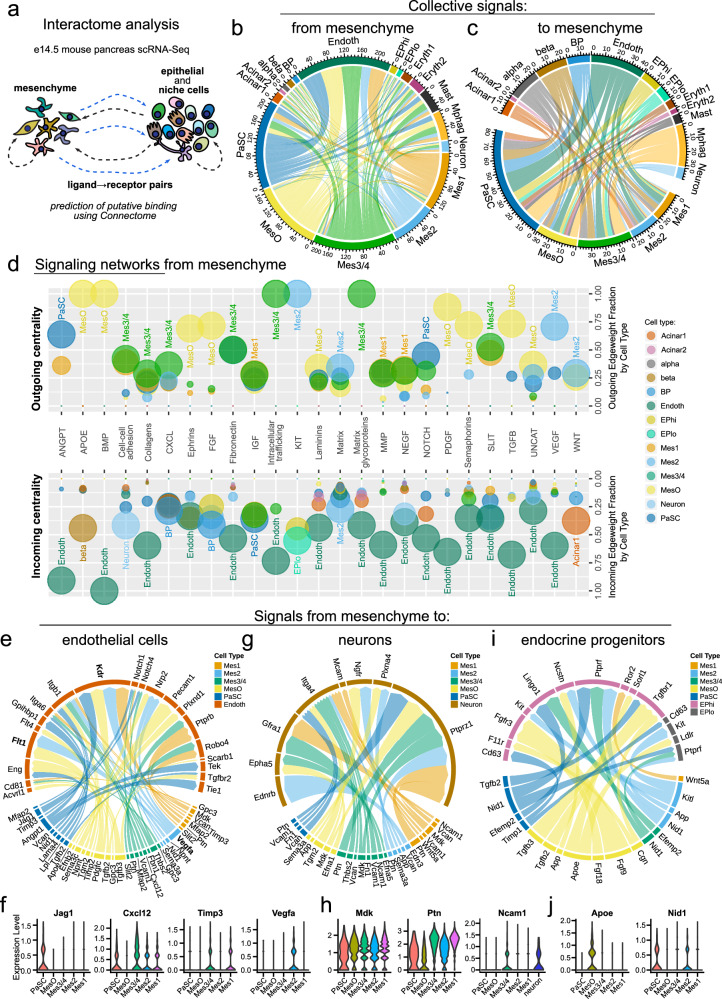
Intercellular interaction maps of pancreatic mesenchyme subtypes. **a** Schematic illustration of interactome analysis of the e14.5 mouse pancreas. Connectome interaction maps showing the predicted cellular communication from mesenchymal subtypes to other cells of the developing pancreas (**b**) and from the other cells to mesenchymal subtypes (**c**). Lines show mesenchymal-expressed ligands computationally linked to receptors expressed in other cell types. Line colors indicate the ligand cell type of origin. **d** Signaling networks from mesenchymal subtypes to other cell types are shown by dot plot visualization of centrality analysis. Selected signaling networks (x-axis) are shown, with the upper outgoing graphs showing the expression of network ligands in mesenchymal subtypes and the lower incoming graphs showing corresponding network receptors in other pancreatic cell types. The color of each dot represents the cell type. The edgeweight fraction (y-axis) reflects the weighted use of a network, with values closer to 1 indicating cell types with greater use in relation to other cell types. The in-graph labels indicate the cell types with the highest edgeweight values within each network. The dot size reflects the normalized expression level of ligands (outcoming graph) or receptors (incoming graph) belonging to a network within a cell type. Connectome interaction maps showing the predicted cellular ligand‒receptor communication from mesenchymal subtypes to endothelial cells (**e**), neurons (**g**) and endocrine progenitors (**i**). Line colors indicate the ligand-expressing cell type, and the thickness of the line ends depends on the edgeweight score of each particular end, i.e., a ligand or a receptor score. Violin plots showing normalized expression of selected ligands in mesenchyme subtypes that putatively interact with receptors in endothelial cells (**f**), neurons (**h**), and endocrine progenitors (**j**).

Cell-to-cell communication is critical for the development and maintenance of cells. Thus, we outlined an atlas of potential ligand‒receptor pairs, establishing the interactome among all cell types within the developing e14.5 pancreas (Fig. 6a). Next, we used these data to identify putative signaling pathways originating from mesenchymal subtypes and targeting nonmesenchymal cells. Using Connectome^28^, we assessed the correlated expression of ligand‒receptor pairs between cell types, defining both autocrine and paracrine interactions and generating network visualizations of the interactome (Fig. 6b, c; Dataset 1). This analysis revealed, for example, significant crosstalk between mesenchymal and endothelial cells. We next identified putative intercellular signaling pathways from mesenchymal subpopulations (Fig. 6d) and, inversely, putative signaling pathways targeting mesenchymal subpopulations (Supplementary Fig. 8a). For example, our analysis indicated that Mes2 is a source of provascularization VEGF signaling that targets endothelial cells (Fig. 6d), which supports the putative role of Mes2 in pancreatic blood vessel generation. We also found that mesenchyme subtypes are a source of CXCL signaling that target epithelial and endothelial cells (Fig. 6d). Interestingly, the CXCL12–CXCR4 axis was shown to influence branching and vascularization of the developing pancreas^51,52^. However, the pancreatic mesenchyme has not been established as a Cxcl12 source.

Given that the network analysis revealed specific modes of interaction between mesenchymal subtypes and other cell types, we next focused on specific ligand‒receptor pairs underlying these interactions (Fig. 6e–i, Supplementary Fig. 8b–f). This analysis revealed that mesenchyme subtypes collectively regulated blood vessel formation, stabilization, and survival while also restricting vessel growth via multiple pathways. These include collagen signaling (common to all mesenchymal subtypes) (Supplementary Fig. 8b), *Cxcl12-Itgb1* (Mes3/4) (Fig. 6e, f), *Jag1-Notch1/4* (PaSC) (Fig. 6e, f), and *Angpt1-Tie1/Tek2* (PaSC) (Fig. 6e). Interestingly, *Vegfa*, a well-established proproliferative, prosprouting, and promigratory factor, was exclusively expressed by Mes2 (Fig. 6e, f), and its receptors *Flt1* (*Vegfr1*) and *Kdr* (*Vegfr2*) were among the most highly expressed receptors in endothelial cells (Fig. 6e, Supplementary Fig. 8c), resulting in high predicted interaction scores (Supplementary Fig. 8d). In contrast, Mes1 and Mes3/4 expressed *Timp3* (Fig. 6e, f), which encodes a metalloproteinase that can inhibit the proangiogenic action of Vegfa through interactions with Kdr^53^.

Ligand‒receptor analysis further suggested that mesenchymal cells regulate pancreatic neurogenesis via midkine-PTPRζ (Mdk-Ptprz1) and pleiothropin-PTPRζ (Ptn-Ptprz1) (Fig. 6g, h, Supplementary Fig. 8e), which promote neurite outgrowth, axon guidance, and neuronal survival. Additionally, Mes3/4 expressing Ncam1 may regulate neurogenesis via mesenchymal–neuronal Ncam1–Ncam1 interactions (Fig. 6g, h). Interestingly, neuronal cells also expressed *Gfra1* (Fig. 6g), and the Ncam1–Gfra1 interaction can inhibit Ncam1 neurogenic activity^54^. Consistent with the findings of the GO analysis suggesting cross-talk between MesO and the epithelium, MesO-expressed ligands were found to pair with receptors on bipotent cells (BPs), endocrine progenitors (EPs), and endocrine cells. Examples include Fgf9 signaling to BPs and EPs (Fig. 6i, Supplementary Fig. 8f), Apoe signaling to EPs and endocrine cells (Fig. 6I, j, Supplementary Fig. 8g), and Tgfb2/3 signaling to EPs (Fig. 6i). Among the mesenchymal signaling pathways that target other pancreatic cell types, the top scoring pairs were *Nid1*-*Ptprf* between mesenchymal and epithelial/endocrine cells (Fig. 6I, j, Supplementary Fig. 8f-g) and the integrins-*Sdc4* receptor in BPs (Supplementary Fig. 8f). Together, the computationally predicted intercellular interactions of the individual mesenchyme populations suggest that these subtypes could perform different functions during pancreatic organogenesis. This dataset provides a framework for a functional understanding of mesenchymal subpopulations in pancreatic organogenesis.

### Mesenchyme subtypes in the developing human pancreas

**Fig. 7:**
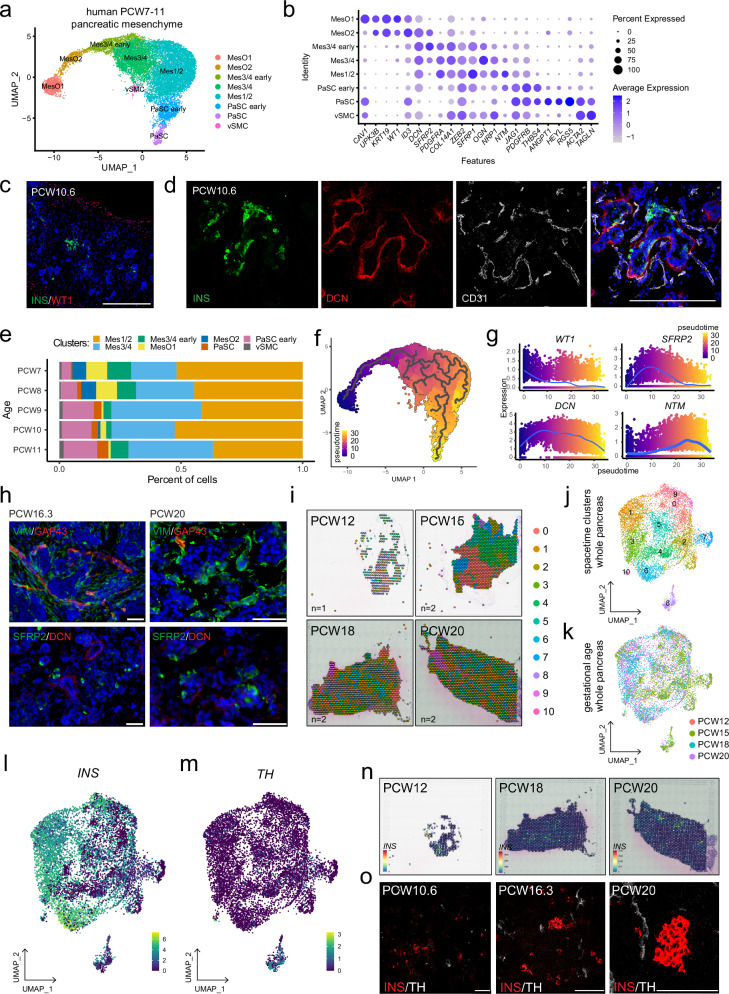
Mesenchymal diversity in the developing human pancreas. **a** Single-cell RNA-sequencing UMAP feature of mesenchyme subclustered from human fetal pancreata at 7–11 PCW (OMIX001616 dataset^43^), with colors featuring mesenchymal subtypes. Clusters of proliferating mesenchymal cells (see Supplementary Fig. 9a) were not included in further analysis. **b** Dot plot of the expression of select murine mesenchymal subtype marker genes in the human PCW 7–11 pancreatic mesenchyme. Each dot represents a gene, with its size indicating the percentage of cells within a cluster expressing the marker (pct. exp.) and its color represents the average expression level (avg. exp.). **c** Representative immunostaining image of a human PCW 10.6 pancreas showing beta cells (INS, green) and WT1+ MesO (red). Nuclei are labeled with DAPI (blue). Scale bar = 200 μm. **d** Representative immunostaining image of a human PCW 10.6 pancreas showing developing beta cells (INS, green), blood vessels (CD31, white) and DCN+ mesenchyme (red). Nuclei are labeled with DAPI (blue). Scale bar = 200 μm. **e** Stack plot depicting the proportional distribution of pancreatic mesenchyme subtypes across different gestational ages (PCW 7–11) in the human fetal pancreas. **f** Monocle3 trajectory feature plot of the PCW 7–11 human fetal pancreas visualizing potential cell fate transitions. The trajectories are denoted by gray branching lines, with the color scale indicating pseudotime. Dark blue signifies the earliest computed timepoint, corresponding to MesO, and yellow indicates the latest computed timepoint, corresponding to PaSCs and the subpopulation of the Mes1/2 cluster. **g** Plots illustrating the dynamics of selected marker gene expression throughout the inferred cell fate trajectory. Dots represent individual cells. **h** Representative images of immunostained human PCW 16.3 and PCW 20 pancreata. The top panels show GAP43+ (red) mesenchyme and VIM+ staining (green) as global mesenchyme markers. The bottom panels show SFRP2+ (green) and DCN+ (red) mesenchyme. Nuclei are labeled with DAPI (blue). Scale bars = 50 μm. **i** Visualization of spatial transcriptomics clusters in human fetal pancreata at PCW 12, 15, 18, and 20 (GSE197317 dataset). Space–time UMAP feature plots of PCW 12, 15, 18, and 20 human fetal pancreata spatial transcriptomics with colors featuring mesenchymal subtypes (**j**) or gestational age (**k**). **l**, **m**. UMAP feature plots of *INS* and *TH* relative expression levels in PCW 12– to 20 human fetal pancreata. **n** Visualization of spatial *INS* expression in human fetal pancreata at PCW 12, 18, and 20. **o** Representative immunostaining of human pancreata PCW 10.6, 16.3, and 20 showing INS (red) and TH (white) expression. Scale bars = 100 μm.

The mesenchymal subtypes we identified spatially coordinated with other niche cells throughout mouse pancreatic development. We next sought to understand whether these subtypes were also present during human pancreatic development. Using a publicly available scRNA-Seq dataset of human fetal pancreatic tissue at postconception week (PCW) 7 to –11^29^, we detected widespread mesenchymal heterogeneity (Fig. 7a, Supplementary Fig. 9a). The identified human pancreatic mesenchymal clusters appeared to be conserved with subpopulations identified in the mouse e14.5 pancreas, as both share multiple markers (Fig. 7b). Two MesO cells were enriched in *WT1*, *CAV1*, *UPK3B* and *KRT19*, whereas Mes3/4 cells expressed *SFRP2* and *OGN*, and the Mes1/2 cluster was enriched in *SFRP1* and *NTM*. Finally, in the developing human pancreas, we identified two PaSC clusters that were enriched for *ACTA2*, *TAGLN*, and *RGS5* transcripts. DCN was among the transcripts detected in a large proportion of the cells of all the subtypes, except for the PaSC cluster.

Next, we performed immunostaining of early-gestation pancreatic tissue at 10.6 weeks, when NGN3 expression is peaking, and islets are highly vascularized and innervated^55–57^. We confirmed the expression of the mesenchymal markers WT1 and DCN (Fig. 7c, d, Supplementary Fig. 9b), with WT1+ cells located outside the pancreas, whereas DCN+ cells were proximal to developing INS+ endocrine cells surrounded by CD31+ endothelial cells. The proportions of interspecies conserved clusters changed over the gestation weeks (Fig. 7e), with diminishing MesO clusters and increasing cell numbers within PaSC clusters. Furthermore, we performed trajectory analysis with Monocle3. This analysis revealed a main branch reaching from MesO through Mes3/4 and Mes1/2 to PaSCs (Fig. 7f), which corroborated the pseudotime inference of the mouse e14.5 pancreas (Fig. 2a–c). The *WT1*, *SFRP2*, *DCN*, and *NTM* expression patterns in the pseudotime analysis corresponded to the pseudotime appearance of the clusters within which they were found (Fig. 7g). *GAP43*, a marker of the mouse Mes1 subtype, seemed to be rarely expressed in the human PCW7-12 pancreas. However, *GAP43* expression slightly increased along the pseudotime (Supplementary Fig. 9d), and we found that its expression was enriched in a trajectory side branch within the Mes1/2 cluster (Fig. 7f, Supplementary Fig. 9e, f), a subpopulation whose gene expression profile was similar to that of the mouse Mes1 subtype. Our scRNA-Seq analysis revealed rare expression of the Mes4 marker *ACE2* in PCW7-11 pancreatic mesenchyme cells. Consistent with this finding, immunofluorescence staining of the PCW10.6 human fetal pancreas revealed few ACE2+ cells, which were primarily localized near INS+ cells (Supplementary Fig. 9c).

We then investigated how mesenchymal subtypes change in the later stages of human pancreas development. At PCW16.3 and PCW20, we found that mesenchymal cells contained a subset of cells that were GAP43 + VIM+ and that SFRP2 and DCN proteins are mostly not coexpressed with each other (Fig. 7h). Analysis of the human fetal pancreatic scRNA-Seq data of PCW12-20^30^ revealed mesenchymal heterogeneity and shifting in cluster proportion over time (Supplementary Fig. 9g-i). Within these data, we detected SFRP2 + DCN+ mesenchymal cells and broader DCN + SFRP- populations (Supplementary Fig. 9j, k). However, at these later stages of pancreas development, mesenchymal populations substantially changed as compared to those of the PCW7–11 pancreas, likely reflecting the progression of the pancreas to an adult-like appearance. Indeed, using spatial transcriptomics of the developing human pancreas at PCW12–20^30^ allowed us to observe organizational changes in mesenchymal subtypes over time (Fig. 7i–k). Focusing on beta cells and neurons, we observed a shift in the localization of these cells over time both at the single-cell transcriptional level and by immunostaining (Fig. 7l–o). Together, these data reveal that there is conservation in mesenchymal heterogeneity between mouse and human pancreatic development.

## Discussion

The influence of mesenchymal cells on epithelial development has been acknowledged over the years and continues to grow in recognition^58–62^. In the pancreas, many groups, including our own, have focused their efforts on investigating the relationships between the mesenchyme and endocrine differentiation during development^6–8,63,64^. This interest has grown in part owing to the urgent need for a steady source of pancreatic beta cells, as rederiving these cells could provide a platform for drug screening or regenerative medicine to treat diseases such as diabetes. However, beyond the increased investigation into mesenchyme‒endocrine interactions, the influence of the mesenchyme in directing the organization and development of other pancreatic cell types, such as nerves and blood vessels, has been largely overlooked. These other cell types, nerves and blood vessels, also provide essential signals that foster endocrine development; therefore, understanding their development and patterning is highly important.

In this study, using our previously described scRNA-seq^16^, we decoded the heterogeneity of the embryonic pancreatic mesenchyme by further characterization of five transcriptionally discrete subtypes, Mes1, Mes2, Mes3/4, MesO, and PaSCs, present at e14.5. The proposed subtypes agree with those found by another group^17^ on the basis of their main markers, although they were named differently. Moreover, in our subclustering approach, we did not include clusters of proliferating mesenchyme. However, in addition to transcriptional profiling, we spatially mapped mesenchyme subtypes before analysis of the ligand‒receptor interactome, providing deeper insight into the organization and architecture of the pancreas during organogenesis. We showed that different subtypes of mesenchyme existed and that these subtypes differed not only in their molecular signatures but also in their localization in the developing pancreas. Together, the results of this study suggest that mesenchyme subtypes have unique functions in the growing pancreas. Importantly, as mesenchymal cells impart cues not only to the developing epithelium but also to innervating neurons and endothelial cells, we suggest that these diverse mesenchyme subtypes interact with other niche components.

We found that the Wt1+ MesO mesenchymal subtype forms a peripheral mesothelium outlining the pancreas between e12.5 and e16.5 in mice, corroborating previous findings^43^. The pancreatic mesothelium and its ancestor, the splanchnic mesoderm, are the original sources of the splenopancreatic and pancreatic mesenchyme at early stages of pancreatic development^43,65,66^, with ~58.6% and ~34.7% mesenchymal cells at e12.5 and e14.5, respectively, being progeny of Wt1-expressing ancestors^43^. However, whether MesO still gives rise to the pancreatic mesenchyme during secondary transition was unknown. In this work, using tamoxifen-induced lineage tracing, we found that Wt1-expressing pancreatic mesothelial cells at e12.5 could still give rise to mesenchymal cells from different subtypes present at e14.5.

In addition to showing the role of MesO in the generation of new mesenchymal cells during the secondary transition, our investigation outlined and characterized the global transcriptome, localization, ontogeny, and interactome of the MesO peripheral mesothelium, which could be used to further investigate other ligands expressed from this subtype and how they influence pancreatic development. This is crucial, as an intact Wt1+ mesothelium is necessary for proper growth of the pancreas^43,67^. We found that MesO was the main source of FGF ligands, including *Fgf9* and *Fgf10*, in the e14.5 pancreas, which might promote the proliferation of mesenchymal and epithelial cells expressing FGF receptors. Indeed, MesO-derived FGF9 was recently shown to be crucial for the development of the pancreatic mesenchyme^45^, whereas FGF10 is known for its ability to regulate epithelial growth^44^. Through GO and interactome analyses, we also revealed that MesO ligands, including FGF, Apoe, and Tgfb family members, paired with endocrine progenitor and beta cell receptors, suggesting that MesO acts upon developing endocrine progenitors and beta cells during the early stages of their induction and development. The MesO–endocrine interaction might be counterintuitive, as the spatial distance between MesO and endocrine cells is prominent; however, this finding is in line with previous studies, which revealed that signals important for EP delamination and migration are derived from the periphery of the pancreas^10^. Thus, our data might be instrumental in exploring novel MesO-derived signals crucial for pancreas development and may prove valuable for recapitulating this process in vitro.

A small subtype of mesenchyme, Mes3/4, was found to be localized proximal to developing islets. This subtype expressed cues for neurogenesis and axon guidance, including *Mdk*, *Ptn*, and *Ncam1*^68^. We hypothesize that the close association between Mes3/4 and islets may act as a bridge, providing cues for innervating neurons to guide them toward islets. This bridge might be further supported by the small fraction of Mes3/4 cells we identified to be closely associated with neuronal processes. Future studies should investigate the role of Mes3/4 in neurite outgrowth assays to determine the functional capacity of Mes3/4 in neural guidance. However, these coculture assays could prove challenging, as sorting Mes3/4 from a litter of embryos yields only ~100 cells. Alternatively, genetic ablation experiments might provide more insights into the function of Mes3/4. However, Mes3/4 could furnish other support, especially as we identified this subtype as a significant source of collagens, cell‒cell interaction signaling network ligands, and vascularization-regulating ligands such as *Cxcl12* and *Timp3*. Interestingly, the Mes3/4 marker Ace2 is involved in angiogenesis and vascularization^69^.

Whereas the interactome analysis demonstrated a general role for the mesenchyme in signaling to endothelial cells, VEGF signaling to endothelial cells was observed exclusively from Mes2. Given the proximity of Mes2 cells to blood vessels, combined with the functions predicted by GO and interactome analyses, these data suggest that the Mes2 mesenchyme may play a crucial role in establishing the endocrine‒endothelial axis. Tight regulation of Vegfa during pancreas development was shown to be crucial for proper endothelial plexus remodeling into a functional vessel network^63^, a process that can also be regulated by the Ptn–Kdr interaction (Fig. 6e)^70^, with *Ptn* expressed by all mesenchymal subtypes (Fig. 6h). At e14.5, the Mes2 subtype expressed markers of the splenopancreatic mesenchyme, such as *Nkx2-5*, and we observed strong Nkx2-5 protein localization in the dorsal pancreas, spleen, and duodenum at this stage. Nkx2-5+ mesenchyme cells were shown to regenerate the splenic stromal microenvironment in the spleen^71^ and support endocrine development in the murine pancreas^72^.

Together, the results of our study delineated the transcriptomes of heterogeneous mesenchyme and support cell types, demonstrating how they interact with other niche cells to pattern the pancreas during development. Using multiple timepoints spanning pancreatic development, we found that mesenchyme subtypes coordinate with other cell types over time and can be found at the expected developmental positions. In-depth characterization of pancreatic cell heterogeneity is likely to facilitate efforts to engineer and sustain functional human islets or pancreatic organoids over extended durations, thereby addressing critical knowledge gaps in the regenerative medicine field.

### Limitations of the study

Our study revealed that the pancreatic mesenchyme is diverse and that these cells coordinate geometrically over time with other cell types. Expanding this knowledge to determine the functional consequences of these cell types, especially in relation to their localization, is critical. To do this, sorting, ablation, and transplantation studies are needed at specific embryonic timepoints to clearly show that different subtypes of mesenchyme are needed at specific times and locations. Our single-cell dataset at e14.5 was composed of 6637 mesenchymal cells; the number of unsupervised clusters depended on the number of cells sequenced, and thus, there may be a greater diversity of mesenchyme subtypes with rare populations, necessitating higher throughput analyses. Our work illustrates the major subtypes of the pancreatic mesenchyme, but mining for these rare subtypes will be of interest. We mapped the localization of mesenchyme subtypes with specific cell types at the protein level. While we validated select markers of mesenchyme subtypes against other markers of the same subtype or markers of different subtypes to form initial hypotheses about their fixed spatial distribution and interactions, it is possible that a marker might be expressed in cells belonging to other subtypes or, in the case of secreted proteins, could also be transported to cells of other subtypes. Moreover, these subtypes may coordinate with many other cell types in the niche. It will be interesting to expand this analysis to the transcriptional level and perform spatial transcriptomics throughout a time course. Finally, identifying the signals that drive the geometric coordination of cell types during pancreatic organogenesis would be interesting.

## Supplementary information

Supplementary Information

Movie 1

Movie 2

Dataset 1

## Data Availability

The accession number for the raw data reported in this manuscript is GSE100622, which was first described in Scavuzzo et al.^16^.
