# Supplementary material for: Pancreatic organogenesis mapped through space and time
Source: Exp Mol Med. 2025 Jan 8;57(1):204–20. doi: 10.1038/s12276-024-01384-y (PMC11799519; doi:10.1038/s12276-024-01384-y)
Supplement: Supplementary file 1 — Supplementary Information [file 12276_2024_1384_MOESM1_ESM.pdf]

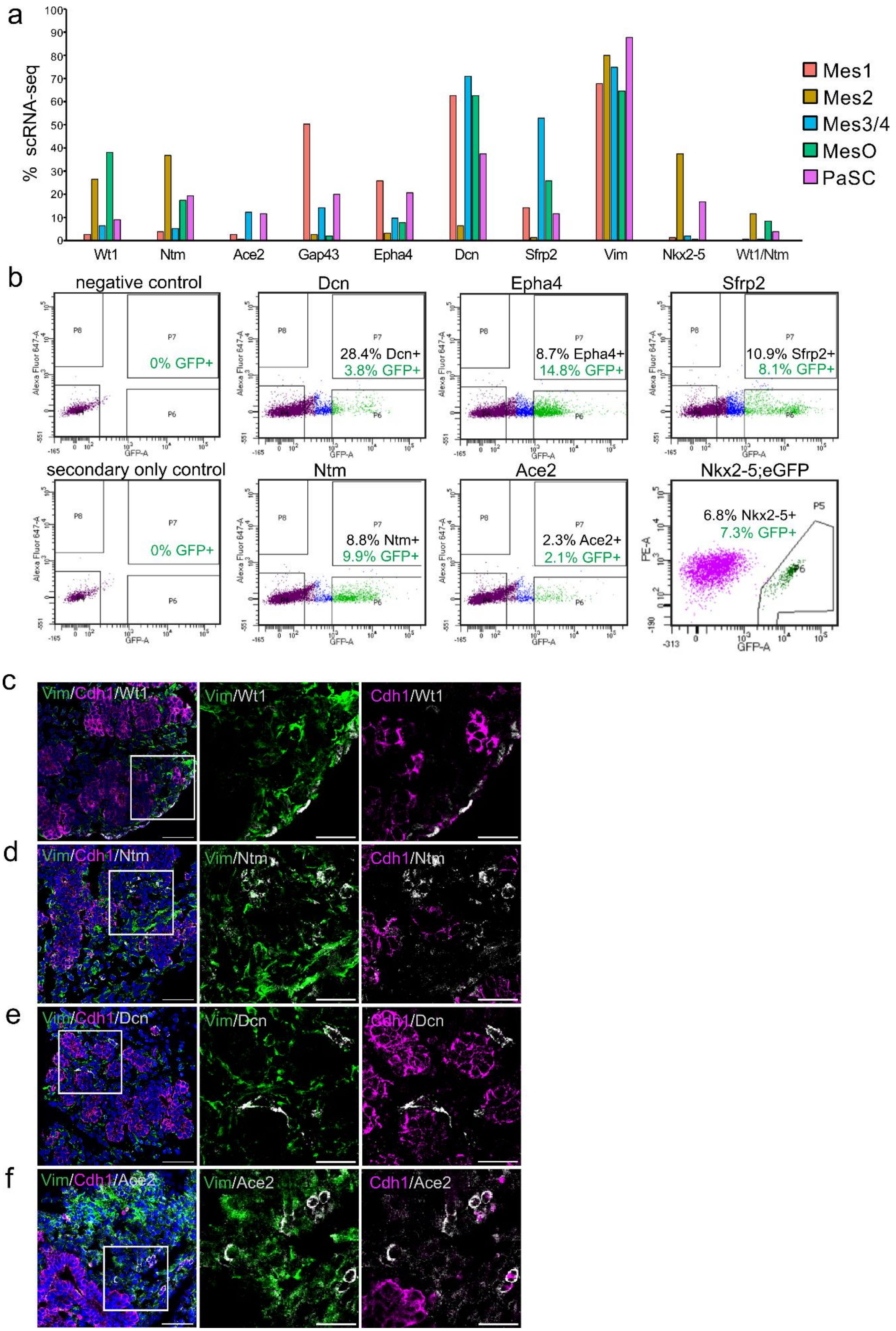

a
% scRNA-seq
Mes1
Mes2
Mes3/4
MesO
PaSC
Wt1
Ntm
Ace2
Gap43
Epha4
Dcn
Sfrp2
Vim
Nkx2-5
Wt1/Ntm
b
negative control
0% GFP+
Dcn
28.4% Dcn+
3.8% GFP+
Epha4
8.7% Epha4+
14.8% GFP+
Sfrp2
10.9% Sfrp2+
8.1% GFP+
secondary only control
0% GFP+
Ntm
8.8% Ntm+
9.9% GFP+
Ace2
2.3% Ace2+
2.1% GFP+
Nkx2-5;eGFP
6.8% Nkx2-5+
7.3% GFP+
Alexa Fluor 647-A
GFP-A
PE-A
c
Vim/Cdh1/Wt1
Vim/Wt1
Cdh1/Wt1
d
Vim/Cdh1/Ntm
Vim/Ntm
Cdh1/Ntm
e
Vim/Cdh1/Dcn
Vim/Dcn
Cdh1/Dcn
f
Vim/Cdh1/Ace2
Vim/Ace2
Cdh1/Ace2

Supplementary Figure 1. Subtype specific marker validation.

a. Quantification based on single-cell RNA-sequencing data of percentage of cells in each mesenchyme subtype expressing genes shown on the x-axis.

b. Flow cytometry analysis of the e14.5 mouse tissue for candidate markers Dcn, Epha4, Sfrp2, Ntm, Ace2, and Nkx2-5-cre;Rosa-mTmG (Nkx2-5-eGFP). In black text shows the percentage of all pancreatic cells expressing candidate markers as found through single-cell RNA-sequencing; in green text shows the percentage of all pancreatic cells expressing candidate markers through flow cytometry readout by GFP signal. On the left, negative controls are shown, i.e. unstained, wildtype cells (top) and cells stained only with secondary antibodies (bottom).

c-f. Representative immunostainings of Wt1+ (c; MesO marker), Ntm+ (d; Mes2 marker), Dcn (e; Mes1, Mes3/4, MesO marker), and Ace2+ mesenchyme (f) cells, in white, in mouse e14.5 pancreata in relation to epithelium (Cdh1+, in magenta) and mesenchyme cells (Vim+, in green). Scale bars = 50 µm (left), 25 µm (middle and right magnifications of squared regions of left images).

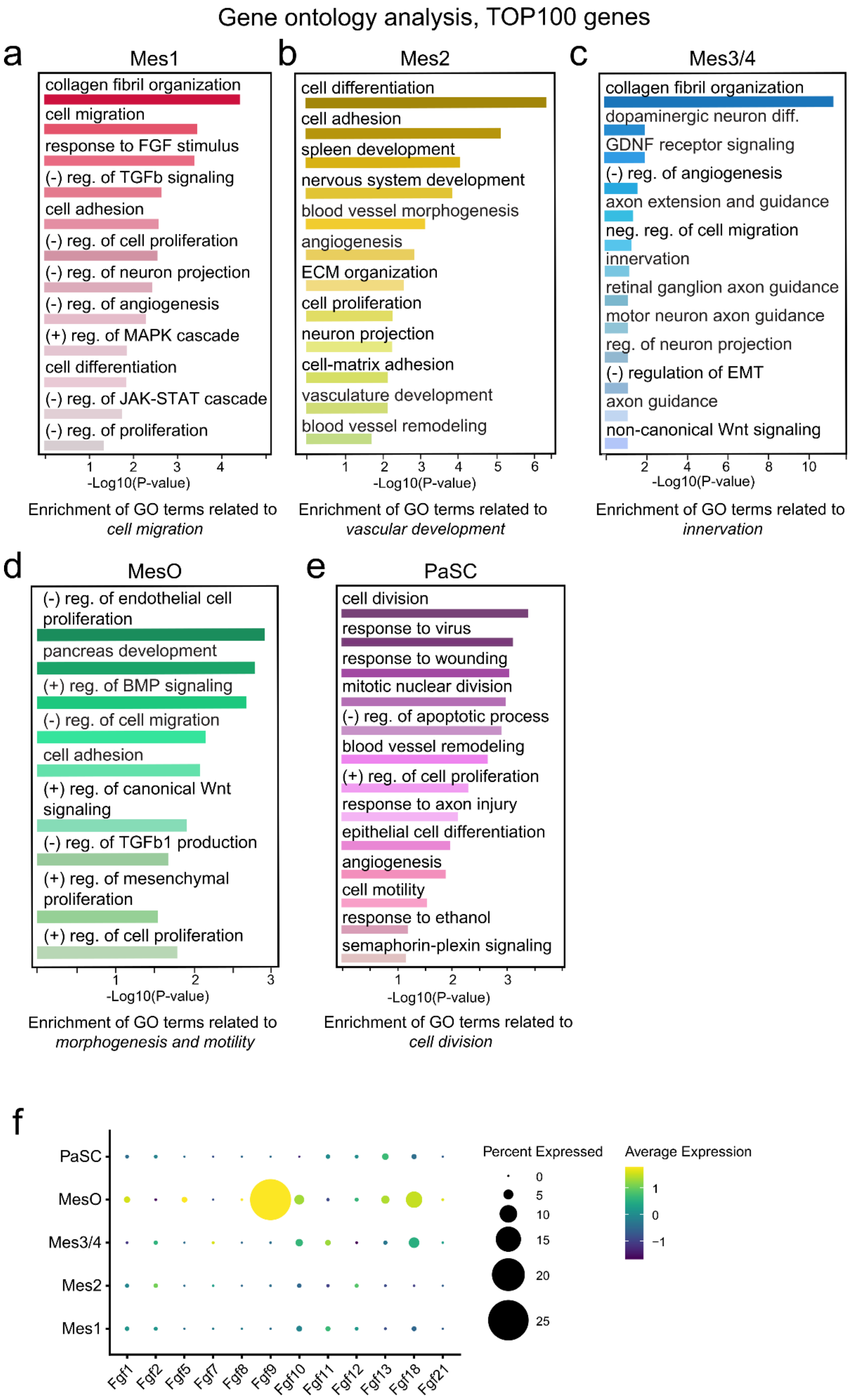

Gene ontology analysis, TOP100 genes
a
Mes1
collagen fibril organization
cell migration
response to FGF stimulus
(-) reg. of TGFb signaling
cell adhesion
(-) reg. of cell proliferation
(-) reg. of neuron projection
(-) reg. of angiogenesis
(+) reg. of MAPK cascade
cell differentiation
(-) reg. of JAK-STAT cascade
(-) reg. of proliferation
1 2 3 4
-Log10(P-value)
Enrichment of GO terms related to *cell migration*
b
Mes2
cell differentiation
cell adhesion
spleen development
nervous system development
blood vessel morphogenesis
angiogenesis
ECM organization
cell proliferation
neuron projection
cell-matrix adhesion
vasculature development
blood vessel remodeling
1 2 3 4 5 6
-Log10(P-value)
Enrichment of GO terms related to *vascular development*
c
Mes3/4
collagen fibril organization
dopaminergic neuron diff.
GDNF receptor signaling
(-) reg. of angiogenesis
axon extension and guidance
neg. reg. of cell migration
innervation
retinal ganglion axon guidance
motor neuron axon guidance
reg. of neuron projection
(-) regulation of EMT
axon guidance
non-canonical Wnt signaling
2 4 6 8 10
-Log10(P-value)
Enrichment of GO terms related to *innervation*
d
MesO
(-) reg. of endothelial cell proliferation
pancreas development
(+) reg. of BMP signaling
(-) reg. of cell migration
cell adhesion
(+) reg. of canonical Wnt signaling
(-) reg. of TGFb1 production
(+) reg. of mesenchymal proliferation
(+) reg. of cell proliferation
1 2 3
-Log10(P-value)
Enrichment of GO terms related to *morphogenesis and motility*
e
PaSC
cell division
response to virus
response to wounding
mitotic nuclear division
(-) reg. of apoptotic process
blood vessel remodeling
(+) reg. of cell proliferation
response to axon injury
epithelial cell differentiation
angiogenesis
cell motility
response to ethanol
semaphorin-plexin signaling
1 2 3
-Log10(P-value)
Enrichment of GO terms related to *cell division*
f
PaSC
MesO
Mes3/4
Mes2
Mes1
Fgf1 Fgf2 Fgf5 Fgf7 Fgf8 Fgf9 Fgf10 Fgf11 Fgf12 Fgf13 Fgf18 Fgf21
Percent Expressed
0
5
10
15
20
25
Average Expression
1
0
−1

Supplementary Figure 2. Enrichment analysis of mesenchyme subtypes revealed by single-cell RNA-sequencing.

a-e. GO Biological process enrichment analysis of (a) Mes1, (b) Mes2, (c) Mes3/4, (d) MesO, and (e) PaSC for top100 genes enriched in each cluster.

f. Fibroblast growth factor (Fgf) family members RNA expression in mesenchyme subtypes.

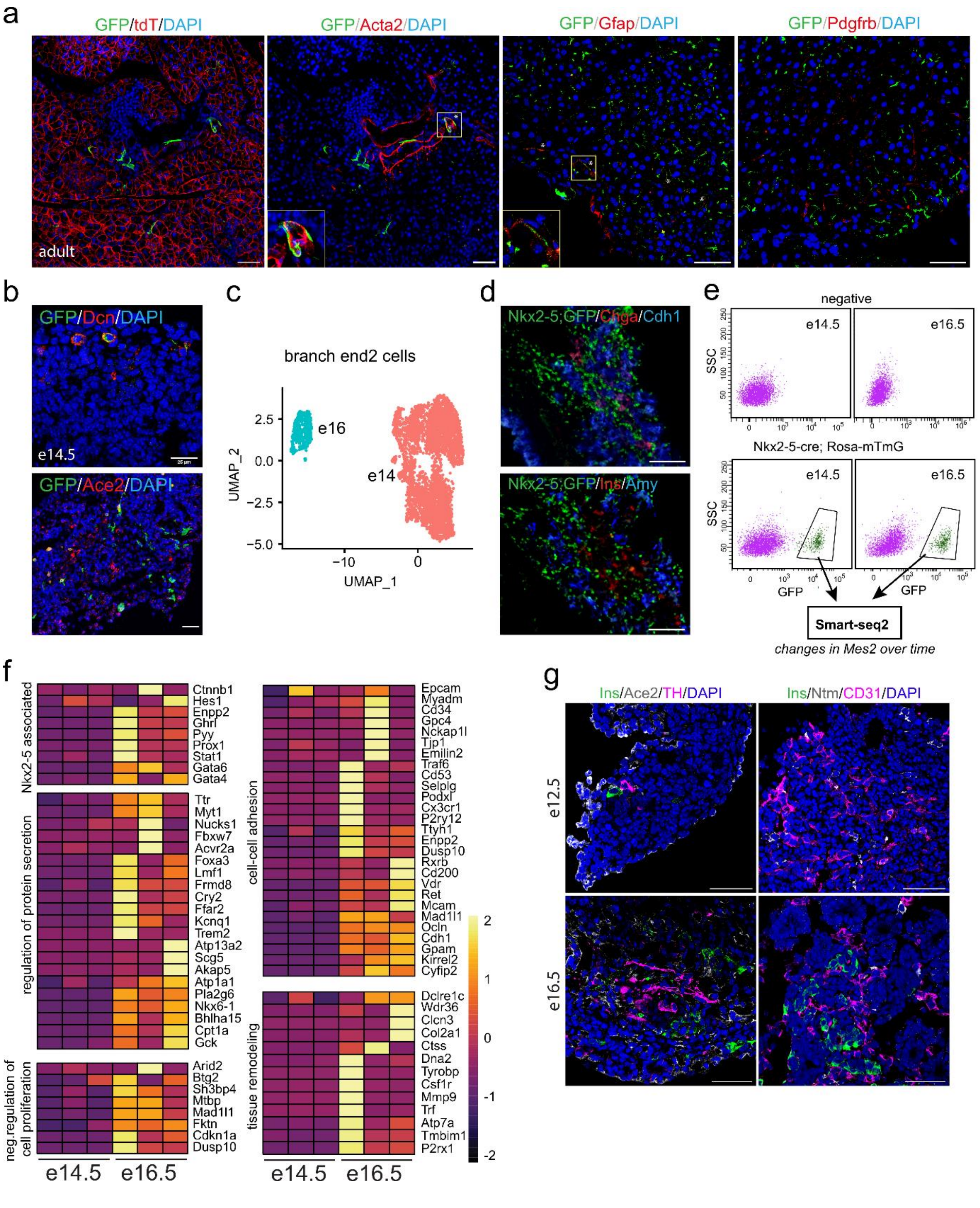
a
GFP/tdT/DAPI
GFP/Acta2/DAPI
GFP/Gfap/DAPI
GFP/Pdgfrb/DAPI
adult
b
GFP/Dcn/DAPI
e14.5
GFP/Ace2/DAPI
c
branch end2 cells
e16
e14
UMAP_2
UMAP_1
d
Nkx2-5;GFP/Chga/Cdh1
Nkx2-5;GFP/Ins/Amy
e
negative
e14.5
e16.5
SSC
Nkx2-5-cre; Rosa-mTmG
GFP
Smart-seq2
changes in Mes2 over time
f
Nkx2-5 associated
Ctnnb1
Hes1
Enpp2
Ghrl
Pyy
Prox1
Stat1
Gata6
Gata4
regulation of protein secretion
Ttr
Myt1
Nucks1
Fbxw7
Acvr2a
Foxa3
Lmf1
Frmd8
Cry2
Ffar2
Kcnq1
Trem2
Atp13a2
Scg5
Akap5
Atp1a1
Pla2g6
Nkx6-1
Bhlha15
Cpt1a
Gck
neg.regulation of cell proliferation
Arid2
Btg2
Sh3bp4
Mtbp
Mad1l1
Fktn
Cdkn1a
Dusp10
e14.5
e16.5
cell-cell adhesion
Epcam
Myadm
Cd34
Gpc4
Nckap1l
Tjp1
Emilin2
Traf6
Cd53
Selplg
Podxl
Cx3cr1
P2ry12
Ttyh1
Enpp2
Dusp10
Rxrb
Cd200
Vdr
Ret
Mcam
Mad1l1
Ocln
Cdh1
Gpam
Kirrel2
Cyfip2
tissue remodeling
Dclre1c
Wdr36
Clcn3
Col2a1
Ctss
Dna2
Tyrobp
Csf1r
Mmp9
Trf
Atp7a
Tmbim1
P2rx1
e14.5
e16.5
g
Ins/Ace2/TH/DAPI
Ins/Ntm/CD31/DAPI
e12.5
e16.5

Supplementary Figure 3. Timelapse dynamics of pancreatic mesenchyme.

a. Representative immunostaining of adult pancreata of Pdgfra-CreERt;Rosa-mTmG mice that were pulsed with tamoxifen at e12.5. On the far left image, tdT (red) stains membranes of all cells except those cells that expressed Pdgfra at e12.5, and progeny of these cells, which express membrane-associated GFP (green). GFP does not overlap extensively with Acta2 (vascular smooth muscle cells marker; pseudocolored red), Gfap (adult PaSCs marker, pseudocolored red) or Pdgfrb (pericyte marker, pseudocolored red) positive cells.
b. Representative immunostaining of e14.5 pancreata showing GFP+ cells (e12.5 Pdgfra+ progeny, in green) partially overlapping with Dcn+ (Mes1, Mes3/4, MesO), and Ace2+ mesenchyme cells, in red. DAPI (in blue) stains nuclei. Scale bar = 25 μm (top), 50 μm (bottom).
c. UMAP reduction of single cell transcriptomes after subclustering e14.5 mesenchyme subtypes Mes1, Mes3/4 and merging with e16.5 single-cell RNA-sequencing of Mes1, Mes3/4.
d. *Top:* Immunostaining of mesenchyme Mes2 subtype marker Nkx2-5;GFP (in green) using Nkx2-5-cre;Rosa-mTmG mice at e14.5 with endocrine cells (Chga, in red) and epithelium (Cdh1, in blue). *Bottom:* Immunostaining of mesenchyme Mes2 subtype marker Nkx2-5 (in green) using Nkx2-5-cre;Rosa-mTmG mice at e14.5 with beta cells (Ins, in red) and acinar cells (Amy, in blue). Scale bars = 100 μm.
e. FACS isolation of Nkx2-5-cre; Rosa-mTmG GFP+ cells for analysis by Smart-seq2. N=3 biological replicates.
f. Heatmap showing select enriched genes in e16.5 Mes2 compared to e14.5. Associated functions by GO analysis are annotated to the left. Expression ranges from low (dark purple) to high (yellow).

g. Representative immunostaining of e12.5 and 16.5 pancreata for mesenchymal subtype-enriched genes Ace2 and Ntm, together with beta cells (Ins+, in green), neurons/alpha cells (TH+), and blood vessels (CD31+), in magenta. Scale bars = 50 μm.

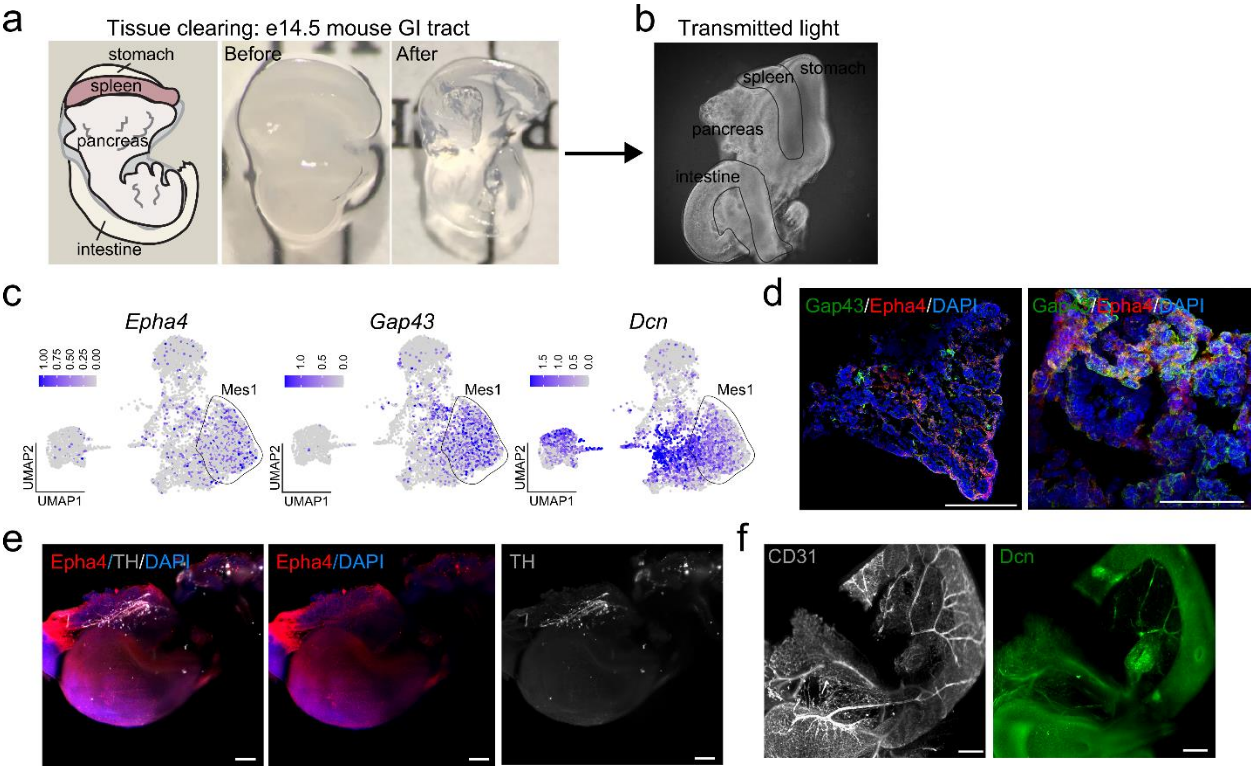

a
Tissue clearing: e14.5 mouse GI tract
stomach
spleen
pancreas
intestine
Before
After
b
Transmitted light
spleen
stomach
pancreas
intestine
c
Epha4
Gap43
Dcn
Mes1
UMAP2
UMAP1
d
Gap43/Epha4/DAPI
e
Epha4/TH/DAPI
Epha4/DAPI
TH
f
CD31
Dcn

Supplementary Figure 4. Light-sheet imaging of optically cleared e14.5 tissue reveals Mes1 subtype localization.

a. Optically clearing of e14.5 GI tract. Tissues were dissected from the esophagus to the duodenum to ensure inclusion of the entire pancreatic region and surrounding niche, before hydrogel formation and breaking up of lipid bilayers through electrophoresis with detergents.
b. Optically cleared e14.5 GI tract in light-sheet by transmitted light.
c. Single-cell RNA-sequencing UMAP feature plots showing mesenchymal expression of *Gap43*, *Epha4* and *Dcn* in Mes1 cluster with dark blue showing high expression and gray showing no expression. The circles outline specific clusters.
d. Immunostaining of Mes1 markers Gap43 (in green) and Epha4 (in red) in 2D sections of embryonic pancreas tissue at e14.5. Nuclei are marked by DAPI in blue. Scale bars = 50 μm.
e. Light-sheet images of optically cleared, immunostained 3D mouse e14.5 pancreas for mesenchymal marker Epha4 (in red) with neurons (TH, in white). Nuclei are marked by DAPI in blue. Scale bars = 200 μm.
f. Light-sheet images of optically cleared, immunostained 3D mouse e14.5 pancreas for Mes1 (Dcn in green) and vasculature (CD31, in white). Scale bars = 200 μm.

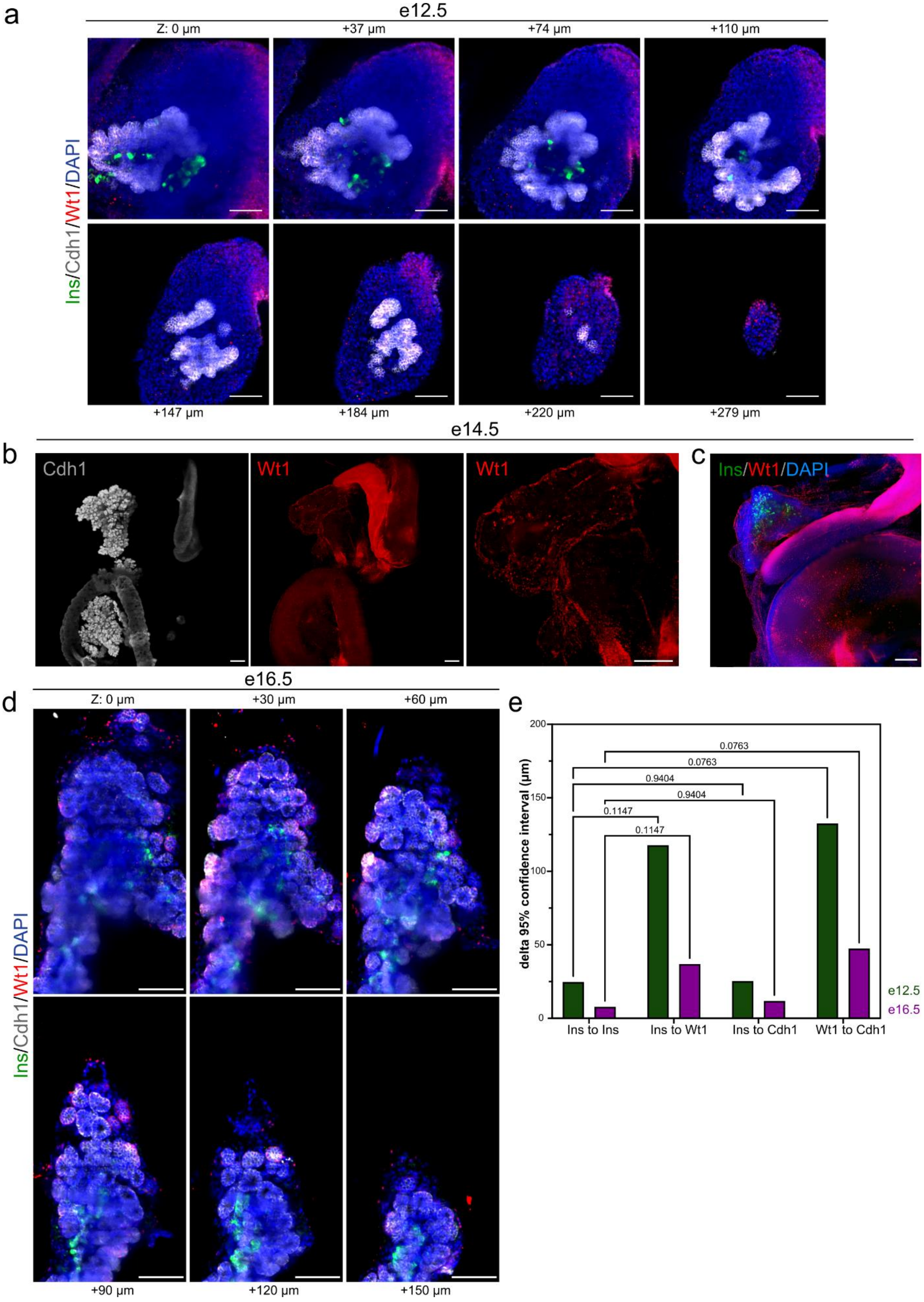
a
e12.5
Z: 0 µm
+37 µm
+74 µm
+110 µm
Ins/Cdh1/Wt1/DAPI
+147 µm
+184 µm
+220 µm
+279 µm
b
e14.5
Cdh1
Wt1
Wt1
c
Ins/Wt1/DAPI
d
e16.5
Z: 0 µm
+30 µm
+60 µm
Ins/Cdh1/Wt1/DAPI
+90 µm
+120 µm
+150 µm
e
delta 95% confidence interval (µm)
200
150
100
50
0
0.0763
0.0763
0.9404
0.9404
0.1147
0.1147
Ins to Ins
Ins to Wt1
Ins to Cdh1
Wt1 to Cdh1
e12.5
e16.5

Supplementary Figure 5. Light-sheet imaging of optically cleared e12.5-e14.5 mouse tissue reveals MesO subtype localization in relation to pancreatic epithelium and beta cells.

a. Optical section images of wholemount 3D light-sheet images of mouse e12.5 pancreas stained for mesenchymal marker Wt1 (in red), epithelium (Cdh1, in white) and beta cells (Ins, in green). Numbers above subsequent section images denote Z-dimension distance shift from the first image. DAPI marks nuclei. Scale bars = 100 μm.

b. Single-channel maximum intensity projection images of wholemount mouse e14.5 pancreas stained for mesenchymal marker Wt1 (in red) and epithelium (Cdh1, in white), corresponding to Fig. 3B. Image on the right shows higher magnification. Scale bars = 200 μm.

c. Maximum intensity projection image of wholemount mouse e14.5 pancreas stained for mesenchymal marker Wt1 (in red) and beta cells (Ins, in green), corresponding to Fig. 3C. Scale bar = 200 μm.

d. Optical section images of wholemount 3D light-sheet images of mouse e16.5 pancreas stained as in (a). Numbers above subsequent section images denote Z-dimension distance shift from the first image. DAPI marks nuclei. Scale bars = 100 μm.

e. Bar plot showing mean of delta 95% confidence intervals of shortest distances between indicated cell types at e12.5 and e16.5. Using the distance between Ins+ cells to other Ins+ cells as a control, we mapped the 95% confidence intervals of spatial relationships, with lower values indicating less variability within biological replicates or a higher likelihood that cells can be found within a specific distance of other cells. As anticipated, Ins+ cells had low intervals, indicating that they are faithfully observed at expected locations relative to other beta cells at both e12.5 and e16.5. In addition, Ins+ beta cells were always found at similar distances to the Cdh1+ epithelium, with no significant difference between the distance between Ins+ cells with each other and Ins+ cells with Cdh1+ cells. Confidence intervals mapped for Wt1+ mesothelial cells with Ins+ cells and the epithelium were not

significantly increased compared to Ins-Ins relationships. Delta was calculated by subtracting upper and lower confidence intervals. P values (Tukey's test post-hoc two-way ANOVA) are shown. N = 3 embryos.

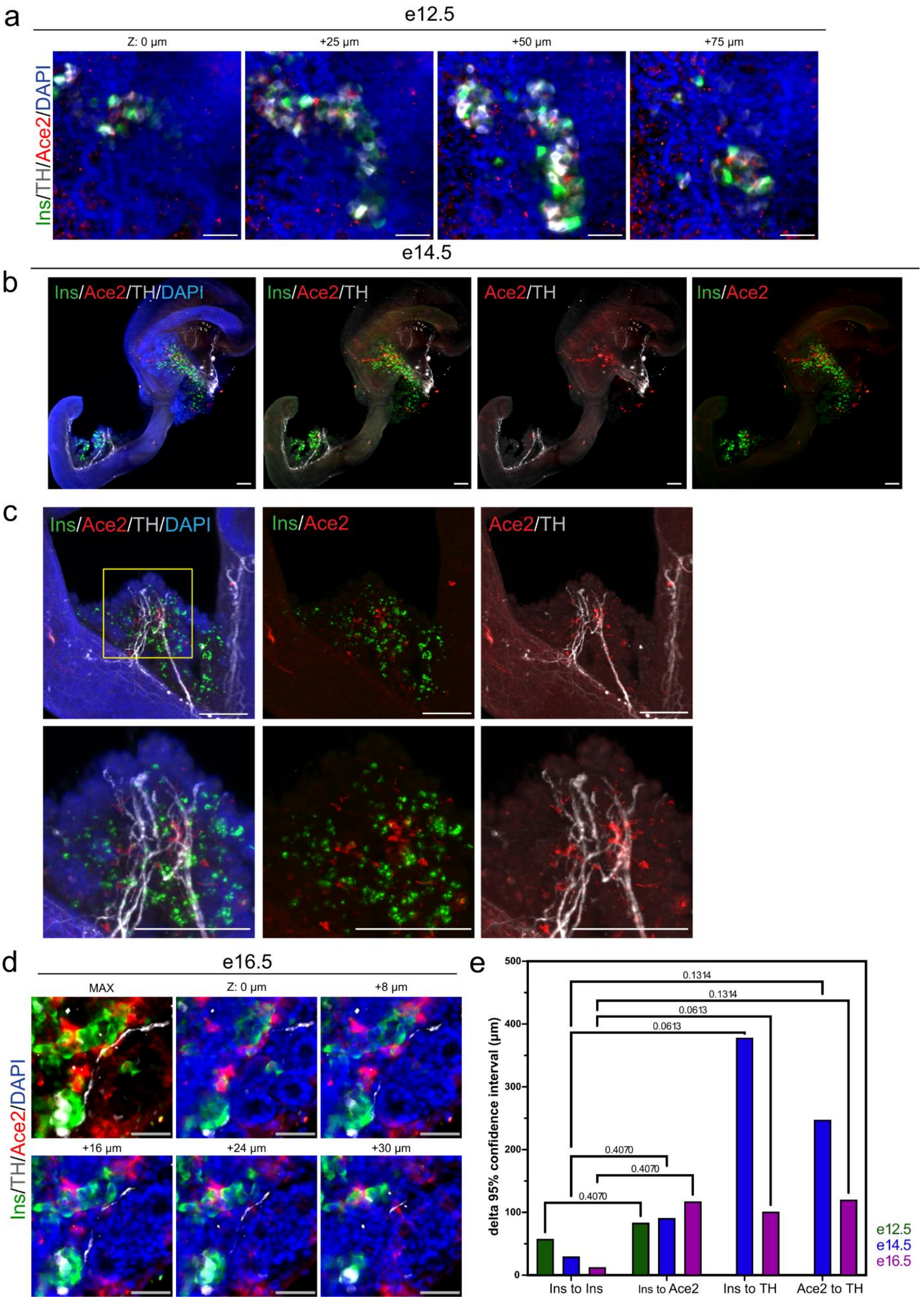
a
e12.5
Z: 0 µm
+25 µm
+50 µm
+75 µm
Ins/TH/Ace2/DAPI
e14.5
b
Ins/Ace2/TH/DAPI
Ins/Ace2/TH
Ace2/TH
Ins/Ace2
c
Ins/Ace2/TH/DAPI
Ins/Ace2
Ace2/TH
d
e16.5
MAX
Z: 0 µm
+8 µm
+16 µm
+24 µm
+30 µm
Ins/TH/Ace2/DAPI
e
delta 95% confidence interval (µm)
500
400
300
200
100
0
0.1314
0.1314
0.0613
0.0613
0.4070
0.4070
0.4070
Ins to Ins
Ins to Ace2
Ins to TH
Ace2 to TH
e12.5
e14.5
e16.5

Supplementary Figure 6. Light-sheet imaging and analysis of Ace2+ pancreatic mesenchyme in spatial relationship with neurons (TH) and beta cells (Ins).

a. Optical section images of wholemount 3D light-sheet images of mouse e12.5 pancreas stained for Ace2+ Mes3/4 mesenchyme (in red), neurons (TH, in white) and beta cells (Ins, in green). At e12.5 we did not find TH+ neuronal processes. Instead, TH marked endocrine alpha-cells. Numbers above subsequent section images denote Z-dimension distance shift from the first image. DAPI marks nuclei. Scale bars = 50 μm.
b. Maximum intensity projection image of wholemount mouse e14.5 pancreas stained for mesenchymal marker Ace2 (in red), neurons (TH in white) and beta cells (Ins in green), corresponding to Fig. 4C. Nuclei are marked by DAPI in blue. Scale bars = 200 μm.
c. Maximum intensity projection image of wholemount mouse e14.5 pancreas stained for mesenchymal marker Ace2 (in red), neurons (TH in white) and beta cells (Ins in green). The yellow square denotes zoomed region shown for each image in the bottom panel. Nuclei are marked by DAPI in blue. Scale bars = 200 μm.
d. Optical section images of wholemount 3D light-sheet images of mouse e16.5 pancreas stained as in (a). Numbers above subsequent section images denote Z-dimension distance shift from the first image. DAPI marks nuclei. Scale bars = 50 μm.
e. Bar plot showing mean of delta 95% confidence intervals of shortest distances between indicated cell types at e12.5, e14.5 and e16.5. Delta was calculated by subtracting upper and lower confidence intervals. P values (Tukey’s test post-hoc two-way ANOVA) are shown. N = 3 embryos.

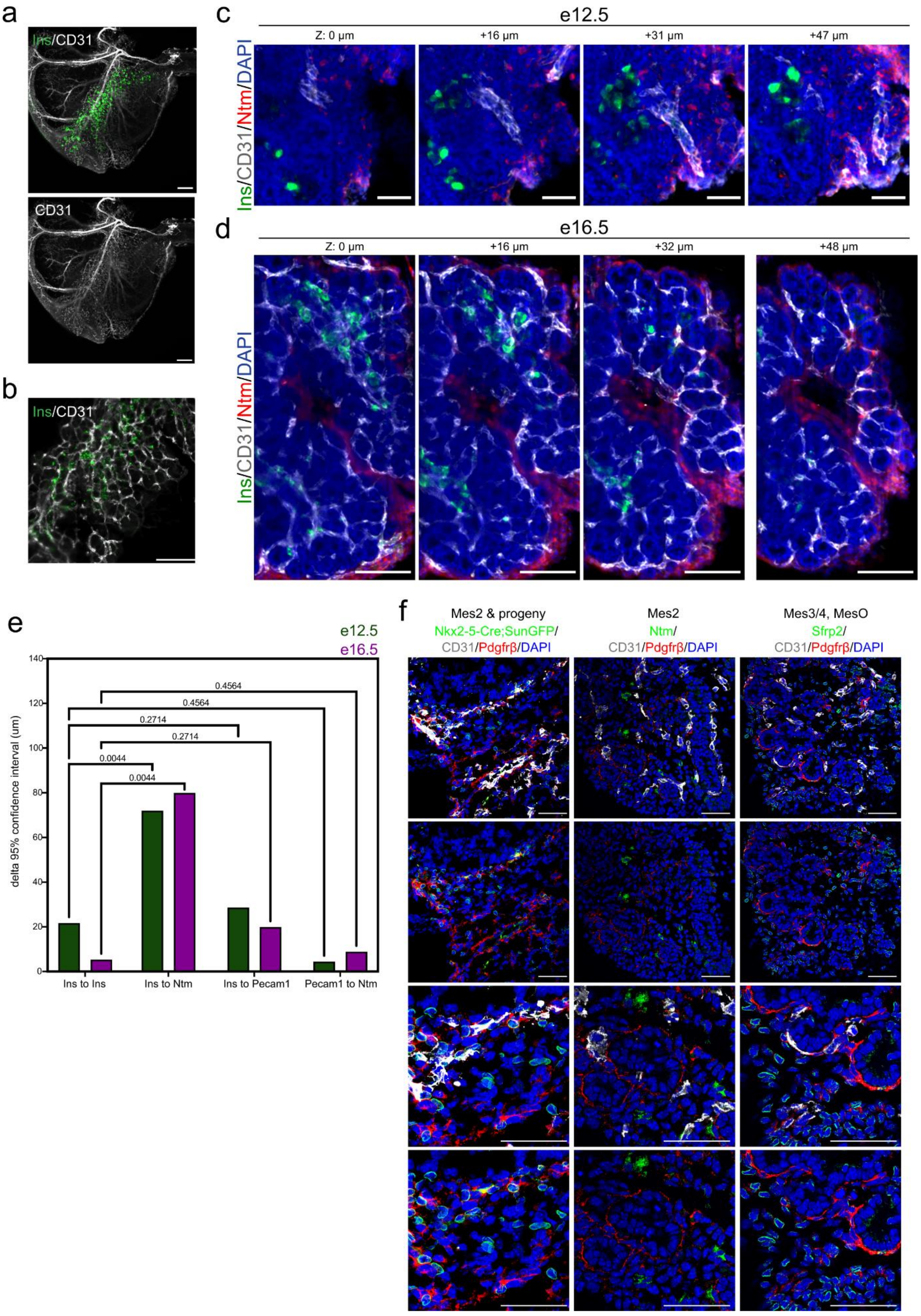

a
Ins/CD31
CD31
b
Ins/CD31
c
e12.5
Z: 0 µm
+16 µm
+31 µm
+47 µm
Ins/CD31/Ntm/DAPI
d
e16.5
Z: 0 µm
+16 µm
+32 µm
+48 µm
Ins/CD31/Ntm/DAPI
e
e12.5
e16.5
delta 95% confidence interval (um)
0.4564
0.4564
0.2714
0.2714
0.0044
0.0044
Ins to Ins
Ins to Ntm
Ins to Pecam1
Pecam1 to Ntm
f
Mes2 & progeny
Nkx2-5-Cre;SunGFP/
CD31/Pdgfrβ/DAPI
Mes2
Ntm/
CD31/Pdgfrβ/DAPI
Mes3/4, MesO
Sfrp2/
CD31/Pdgfrβ/DAPI

Supplementary Figure 7. Mes2 spatial relationship to pancreatic vasculature and beta cells.

a-b. Light-sheet images of optically cleared, immunostained 3D mouse e14.5 pancreas for vasculature (CD31, in white) and beta cells (Ins, in green). Scale bars = 200 μm.

c-d. Optical section images of wholemount 3D light-sheet images of mouse (c) e12.5 and (d) e16.5 pancreata stained for Ntm+ Mes2 mesenchyme (in red), endothelium (CD31, in white) and beta cells (Ins, in green). Numbers above subsequent section images denote Z-dimension distance shift from the first image. DAPI marks nuclei. Scale bars = (c) 50 μm and (d) 100 μm.

e. Bar plot showing mean of delta 95% confidence intervals of shortest distances between indicated cell types at e12.5 and e16.5. Delta was calculated by subtracting upper and lower confidence intervals. P values (Tukey's test post-hoc two-way ANOVA) are shown. N = 3 embryos.

f. Representative immunostainings of Nkx2-5cre (left; Nkx2-5-cre;SunGFP mice; marks Mes2 and their progeny), Ntm+ (middle; Mes2 marker), and Sfrp2+ (right; Mes3/4 marker), in green, in mouse e14.5 pancreata in relation to Pdgfrb+ PaSCs (in red) and CD31+ blood vessels (in white). DAPI (in blue) marks nuclei. Scale bars = 50 μm. Two bottom rows are magnifications of two top rows.

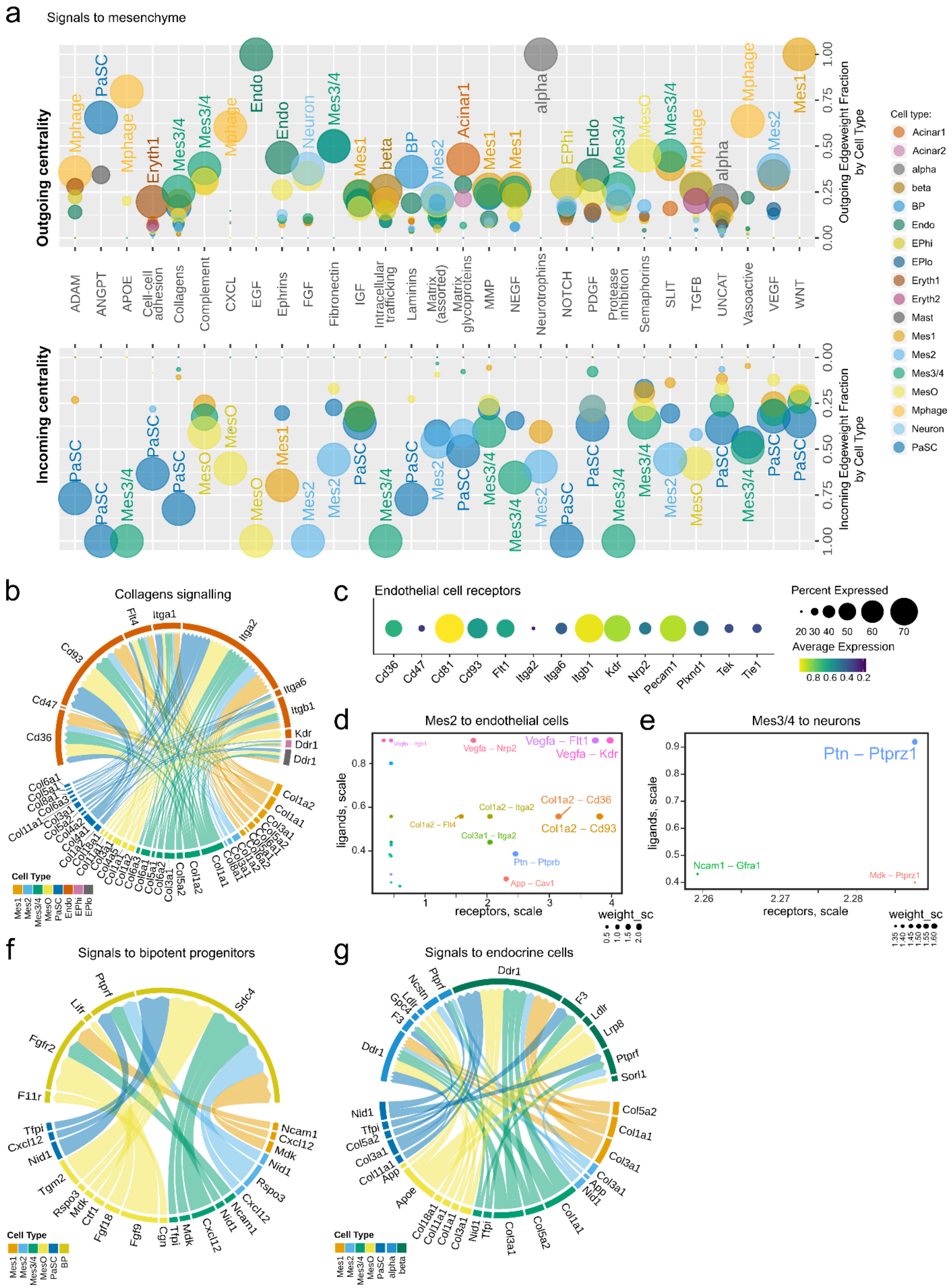
a
Signals to mesenchyme
Outgoing centrality
Outgoing Edgeweight Fraction by Cell Type
Incoming centrality
Incoming Edgeweight Fraction by Cell Type
ADAM
ANGPT
APOE
Cell-cell adhesion
Collagens
Complement
CXCL
EGF
Ephrins
FGF
Fibronectin
IGF
Intracellular trafficking
Laminins
Matrix (assorted)
Matrix glycoproteins
MMP
NEGF
Neurotrophins
NOTCH
PDGF
Protease inhibition
Semaphorins
SLIT
TGFB
UNCAT
Vasoactive
VEGF
WNT
0.00
0.25
0.50
0.75
1.00
Cell type:
Acinar1
Acinar2
alpha
beta
BP
Endo
EPhi
EPlo
Eryth1
Eryth2
Mast
Mes1
Mes2
Mes3/4
MesO
Mphage
Neuron
PaSC
b
Collagens signalling
Cell Type
Mes1
Mes2
Mes3/4
MesO
PaSC
Endo
EPhi
EPlo
c
Endothelial cell receptors
Cd36
Cd47
Cd81
Cd93
Flt1
Itga2
Itga6
Itgb1
Kdr
Nrp2
Pecam1
Plxnd1
Tek
Tie1
Percent Expressed
20 30 40 50 60 70
Average Expression
0.8 0.6 0.4 0.2
d
Mes2 to endothelial cells
Vegfa – Flt1
Vegfa – Kdr
Vegfa – Nrp2
Col1a2 – Cd36
Col1a2 – Cd93
Col1a2 – Itga2
Col1a2 – Flt4
Col3a1 – Itga2
Ptn – Ptprb
App – Cav1
ligands, scale
receptors, scale
weight_sc
e
Mes3/4 to neurons
Ptn – Ptprz1
Ncam1 – Gfra1
Mdk – Ptprz1
ligands, scale
receptors, scale
weight_sc
f
Signals to bipotent progenitors
Cell Type
Mes1
Mes2
Mes3/4
MesO
PaSC
BP
g
Signals to endocrine cells
Cell Type
Mes1
Mes2
Mes3/4
MesO
PaSC
alpha
beta

Supplementary Figure 8. Connectome analysis in e14.5 mouse pancreas

a. Signaling networks from non-mesenchymal pancreatic cells to mesenchymal subtypes shown using dot plot visualization of centrality analysis. Selected signaling networks (x - axis) are shown with the upper outgoing showing expression of network ligands in non-mesenchymal cells subtypes and the lower incoming graphs showing corresponding network receptors in mesenchymal subtypes. Dot colors represent cell type. Edgeweight fraction (y-axis) reflects weighted use of a network, with values closer to 1 indicating cell types with higher use as compared to other cell types. In-graph labels indicate cell types with highest edgeweight values within each network. A dot size reflects normalized expression level of ligands (outcoming graph) or receptors (incoming graph) belonging to a network within a cell type.
b. Connectome interaction maps showing the predicted cellular communication from mesenchymal subtypes to endothelial and EP cells via collagens. Line colors indicate ligand-expressing cell type and thickness of line ends depends on edgeweight score of each particular end, i.e. a ligand or a receptor score.
c. Dot plot showing expression of endothelial cell-expressed receptors revealed by Connectome analysis and detected in more than 20% cells.
d. Ligand-receptor analysis scatter plot showing relative expression levels of ligands (y-axis) and their receptors (x-axis) expressed by Mes2 and endothelial cells, respectively. Dot size (weight_sc) reflects a cumulative expression of a ligand-receptor pair.
e. Ligand-receptor analysis scatter plot showing relative expression levels of ligands (y-axis) and their receptors (x-axis) expressed by Mes3/4 and neurons, respectively. Dot size (weight_sc) reflects a cumulative expression of a ligand-receptor pair.

f, g. Connectome interaction maps showing the predicted cellular communication from mesenchymal subtypes to (f) bipotent progenitor and (g) endocrine cells. Line colors

indicate ligand-expressing cell type and thickness of line ends depends on edgeweight score of each particular end, i.e. a ligand or a receptor score.

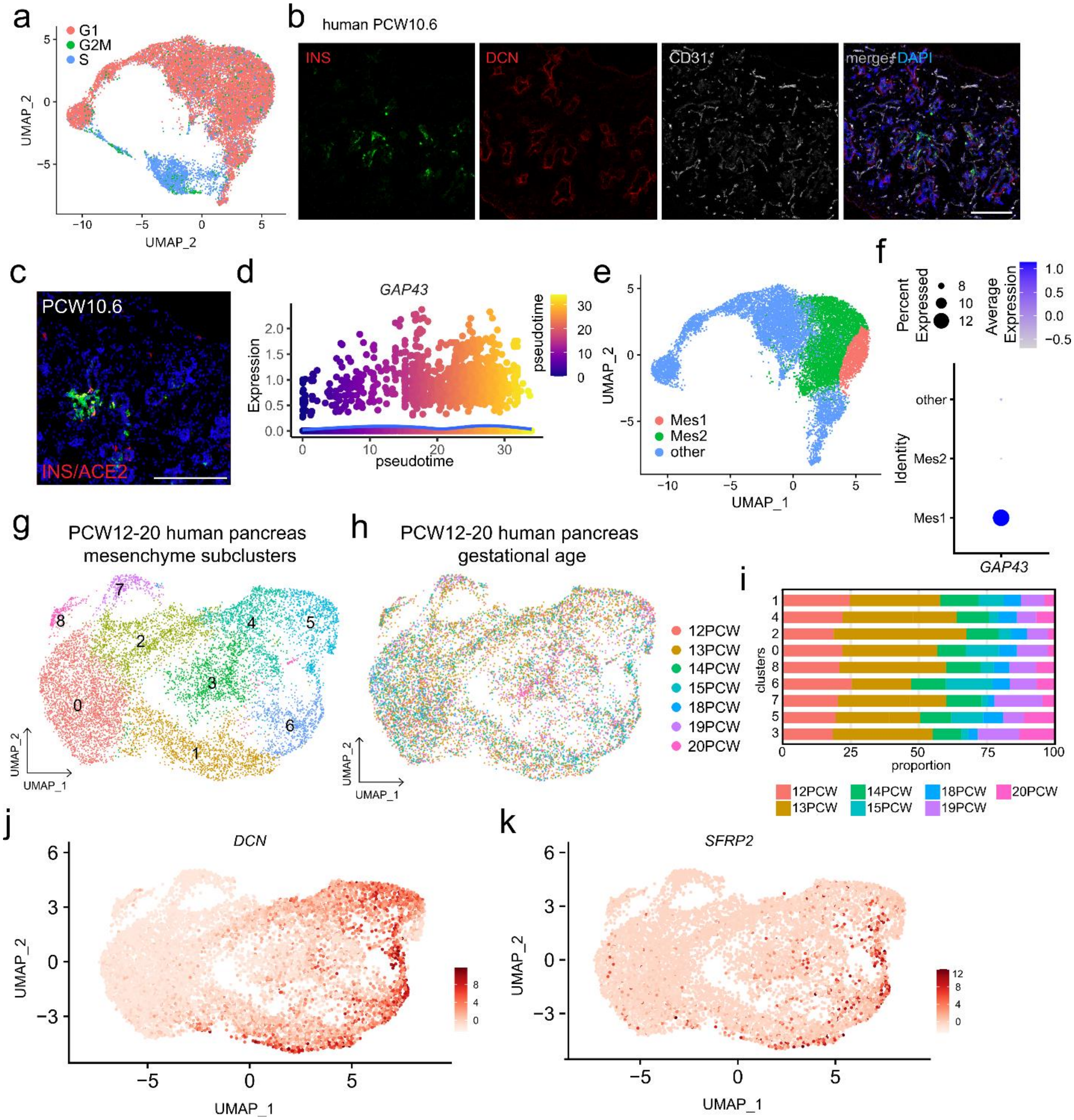
a
G1
G2M
S
UMAP_2
UMAP_2
b
human PCW10.6
INS
DCN
CD31
merge DAPI
c
PCW10.6
INS/ACE2
d
GAP43
Expression
pseudotime
pseudotime
e
Mes1
Mes2
other
UMAP_2
UMAP_1
f
Percent Expressed
Average Expression
other
Mes2
Mes1
Identity
GAP43
g
PCW12-20 human pancreas mesenchyme subclusters
UMAP_2
UMAP_1
h
PCW12-20 human pancreas gestational age
12PCW
13PCW
14PCW
15PCW
18PCW
19PCW
20PCW
UMAP_2
UMAP_1
i
clusters
proportion
12PCW
13PCW
14PCW
15PCW
18PCW
19PCW
20PCW
j
DCN
UMAP_2
UMAP_1
k
SFRP2
UMAP_2
UMAP_1

Supplementary Figure 9. Mesenchyme heterogeneity in human fetal pancreas

a. UMAP feature plot of PCW7-11 human fetal pancreatic mesenchyme showing cell cycle stage of each cell. Separate clusters of G2M (green) and S-phase (blue) cells were removed from the dataset prior to analysis.

b. Immunostaining of human w10.6 pancreas showing developing beta cells (INS, in green) and blood vessels (CD31, in white). DCN+ cells (Mes1, Mes3/4, and MesO) are shown in red. Nuclei are marked by DAPI in blue. Scale bar = 50 μm.

c. Representative Immunostaining of human PCW10.6 pancreas showing developing beta cells (INS, in green) and ACE2+ mesenchyme, in red. Nuclei are marked by DAPI in blue. Scale bar = 200 μm.

d. Plots showing expression changes of *GAP43* expression along pseudotime. Dots represent individual cells.

e. UMAP feature plot of PCW7-11 human fetal pancreatic mesenchyme with identified GAP43+-enriched Monocle3 trajectory branch of Mes1/2 cluster, denoted as Mes1 (in red).

f. Dotplot of *GAP43* expression in Mes1 Monocle3 trajectory branch (Supplementary Fig. 8e - in red) as compared to the rest of Mes1/2 cluster (herein called Mes2; Supplementary Fig. 8e - in green) and other mesenchymal populations (Supplementary Fig. 8d - in blue).

g-h. Single-cell-RNA-sequencing[48] UMAP feature plots of 12 to 20 PCW human fetal pancreata (GSE197064 dataset) with colors featuring (g) mesenchymal subtypes or (h) gestational age.

i. Plot showing proportions of pancreatic mesenchyme subtypes at different gestational ages in human fetal pancreas.

j. UMAP plot of *DCN* relative expression levels in 12 to 20 PCW human fetal pancreatic mesenchyme.

k. UMAP plot of *SFRP2* relative expression levels in 12 to 20 PCW human fetal pancreatic mesenchyme.

Supplementary Table 1. Primary antibody list

Antibody	Catalogue #	Vendor	Dilution
Ace2	PA5-47488	Thermo	1:50
Ace2	ab15348	Abcam	1:100
Acta2	19245	Cell Signal	1:400
Amylase	A8273	Sigma-Aldrich	1:400
aTH	AB152	Millipore	1:200
CD31 (Pecam1)	AF3628	R&D	1:200
Cdh1	AF748	R&D	1:400
Chga	AB15160	Abcam	1:100
Dcn	6D6	DSHB	1:100
Epha4	AF641	R&D	1:50
Gap43	NB3000-143ss	NovusBio	1:500
Gfap	3670	Cell Signal	1:300
GFP	ab13970	Abcam	1:1000
Ins	3014	Cell Signal	1:200
Ins	PA1-26938	Invitrogen	1:100
Ngf	ab52918	Abcam	1:300
Ntm	sc-390941	Santa Cruz	1:100
Pdgfrb	3169	Cell Signal	1:300
Sfrp2	ab137560	Abcam	1:100
Vim	AB5733	Millipore	1:400
Wt1	ab89901	Abcam	1:100

Movie 1. Three-dimensional video showing Ins, Ace2, and TH in the embryonic pancreas and surrounding tissues.

Movie 2. Three-dimensional video showing Ins, Wt1, and Cdh1 in the embryonic pancreas and surrounding tissues
